# A synthetic pregnenolone analog promotes microtubule dynamics and neural development

**DOI:** 10.1186/s13578-022-00923-2

**Published:** 2022-12-01

**Authors:** Viktoryia Kolas, Jose Sandino A. Bandonil, Niaz Wali, Kuo-Chiang Hsia, Jiun-Jie Shie, Bon-chu Chung

**Affiliations:** 1 Institute of Molecular Biology, Academia Sinica, Taipei, Taiwan; 2 Institute of Molecular and Cellular Biology, National Tsing-Hua University, Hsinchu, Taiwan; 3 Institute of Chemistry, Academia Sinica, Taipei, Taiwan; 4 Institute of Biochemical Sciences, National Taiwan University, Taipei, 10617 Taiwan; 5 Chemical Biology and Molecular Biophysics, Taiwan International Graduate Program, (TIGP-CBMB) Academia Sinica, Taipei, 11529 Taiwan; 6 Graduate Institute of Biomedical Sciences, Neuroscience and Brain Disease Center, China Medical University, Taichung, 404 Taiwan

**Keywords:** Cerebellum, P5, Zebrafish, Microtubules, Drug, Neurite

## Abstract

**Background:**

Pregnenolone (P5) is a neurosteroid that promotes microtubule polymerization. It also reduces stress and negative symptoms of schizophrenia, promotes memory, as well as recovery from spinal cord injury. P5 is the first substance in the steroid-synthetic pathway; it can be further metabolized into other steroids. Therefore, it is difficult to differentiate the roles of P5 versus its metabolites in the brain. To alleviate this problem, we synthesized and screened a series of non-metabolizable P5 derivatives for their ability to polymerize microtubules similar to P5.

**Results:**

We identified compound #43 (3-beta-pregnenolone acetate), which increased microtubule polymerization. We showed that compound #43 modified microtubule dynamics in live cells, increased neurite outgrowth and changed growth cone morphology in mouse cerebellar granule neuronal culture. Furthermore, compound #43 promoted the formation of stable microtubule tracks in zebrafish developing cerebellar axons.

**Conclusions:**

We have developed compound #43, a nonmetabolized P5 analog, that recapitulates P5 functions in vivo and can be a new therapeutic candidate for the treatment of neurodevelopmental diseases.

**Supplementary Information:**

The online version contains supplementary material available at 10.1186/s13578-022-00923-2.

## Introduction

Neurosteroids are brain-produced steroids that modulate neuronal activities [1]. All neurosteroids are derived from a single parent steroid, pregnenolone (P5), which enhances memory [2, 3], reduces chronic pain [4], and alleviates depression [5]. P5 also protects rat neurons against damage from prenatal cannabis exposure [6]. Abnormal P5 levels have been observed in schizophrenic patients [7], multiple sclerosis [8], and rats with neuropathic pain [9, 10].

**Fig. 1:**
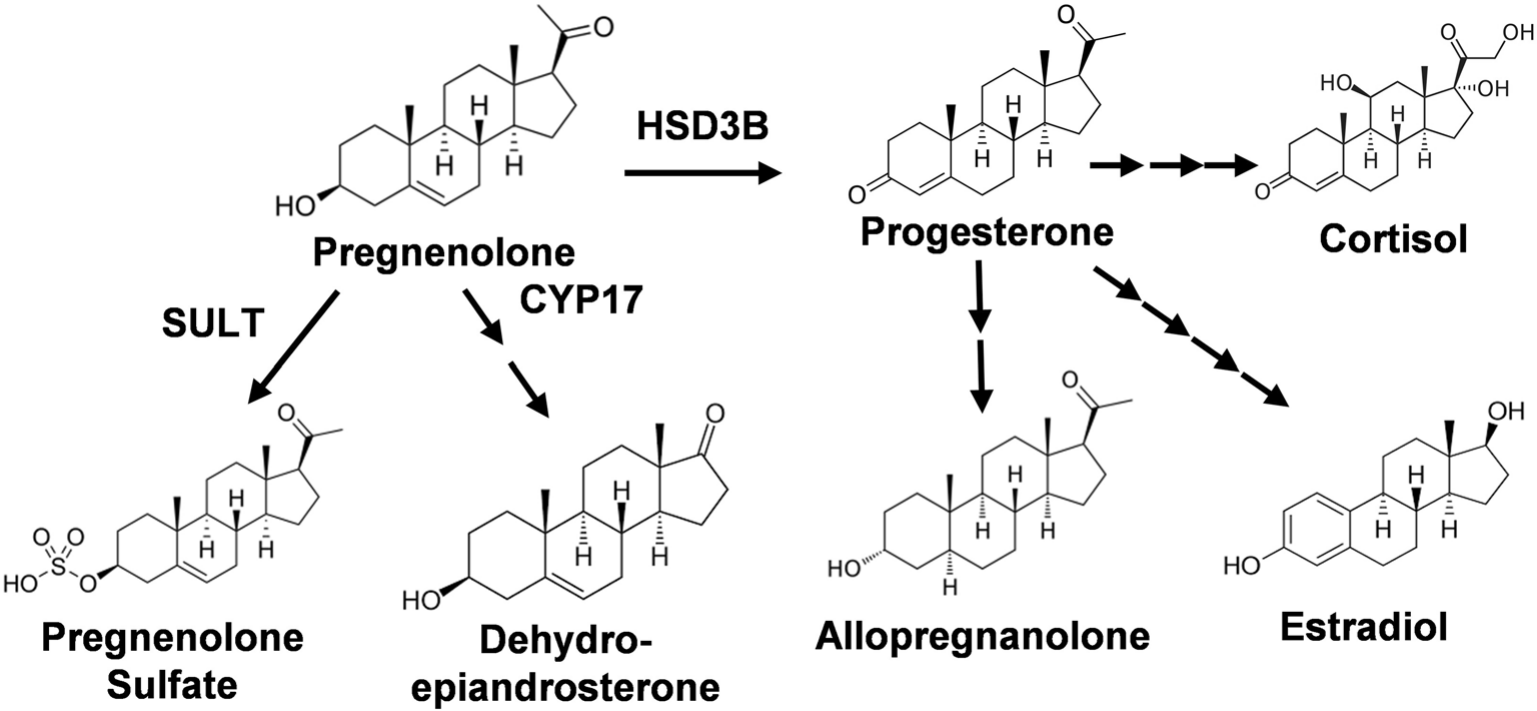
Structures of major P5 metabolites. The structures of major P5 metabolites and the pathways leading to their formation are shown. The first enzymes in the pathways are also labeled. Pregnenolone (P5) can be converted into progesterone by HSD3B (3β-hydroxysteroid dehydrogenase), to pregnenolone sulfate by SULT (sulfotransferase), or to dehydroepiandrosterone by CYP17 (steroid 17α-monooxygenase) followed by other enzymes. Progesterone can be further metabolized into allopregnanolone, cortisol, and estradiol

P5 is produced from cholesterol through the action of CYP11A1 (cytochrome P450scc) [11], which is present in many brain regions [12–15]. P5 is often considered as a parent steroid, because it can be converted to other derivatives such as pregnane, androstane, and sulfated neurosteroids, represented by allopregnanolone, dehydroepiandrosterone, and P5 sulfate, respectively [16, 17] (Fig. 1). Allopregnanolone is associated with neuroprotection and reduction of stress [18]. Dehydroepiandrosterone and its sulfate have roles in neuroprotection and neurite growth [19]. P5 sulfate stimulates learning and memory [20]. 7-Hydroxypregnenolone promotes locomotor activity and long-term memory [20, 21].

Steroids present in the circulation such as progesterone, glucocorticoids and sex steroids can also regulate brain functions [22, 23]. Progesterone regulates brain development, myelination, and neural circuit formation [24]. Glucocorticoids regulate stress response [25]; they also inhibit astrocyte growth in the hippocampus [26]. Estrogen protects against the risk of dementia [27].

The presence of so many P5 derivatives that have brain functions makes it difficult to dissect the active ingredients of neurosteroids. For example, P5 and P5-sulfate both alleviate the symptoms of schizophrenia [28, 29]. However, the sulfate group of P5-sulfate is removed at the blood–brain barrier and therefore its activity may be attributed to P5 [30]. The use of non-metabolizable P5 analogs can help to differentiate the effect of P5 itself from its downstream steroids. These P5 analogs can also be designed to have properties of high bioavailability and low toxicity. Pregnenolone derivatives substituted at C-16 have antiviral activity [31]. Other pregnenolone derivatives are cytotoxic against cancer cell lines [32]. 3-Methoxyl-pregnenolone (meoP5) is a P5 derivative with an effect comparable to that of P5 in neurons [33]. More such derivatives are required to have specific functions in distinct brain regions.

In this study, we screened and characterized P5 derivatives for their effect on neuronal development. The most promising derivative, compound #43 (P5 acetate), promoted neurite extension and induced a change in growth cone morphology in primary cultures of cerebellar granule neurons. It also accelerated the formation of stable microtubule networks in developing zebrafish cerebellum. Thus, compound #43 can be a promising neurosteroid that promotes cerebellar development.

## Results

### Screening of P5 analogs that promote microtubule polymerization

**Fig. 2:**
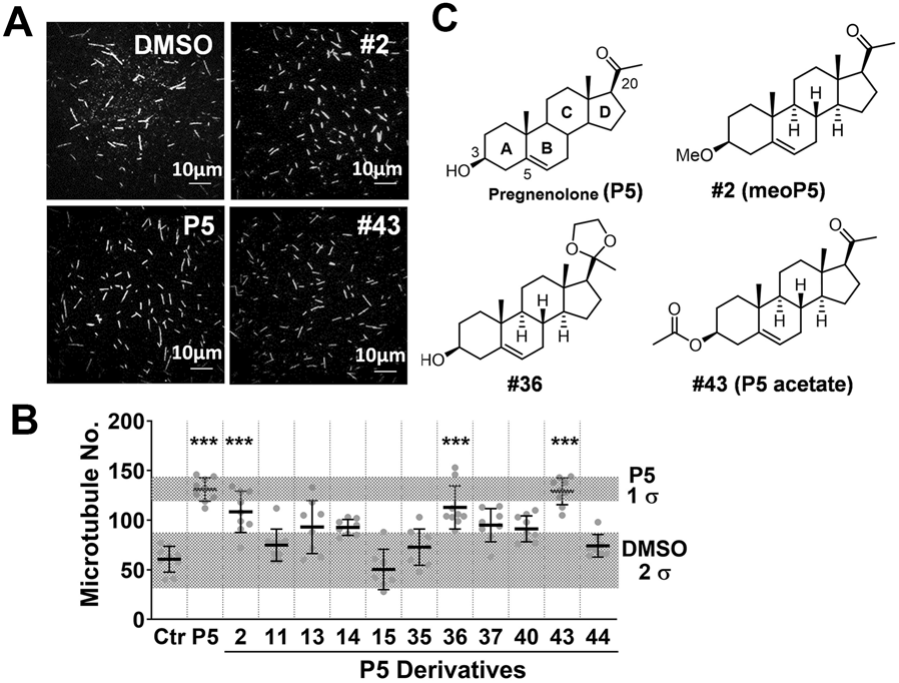
An in vitro screening of P5 derivatives reveals compounds with microtubule polymerization activity similar to P5. **A** Detection of fluorescent microtubules produced by the in vitro microtubule polymerization assay under the TIRF microscopy. Scale bar 10 µm. **B** Screening of compounds that increase microtubule numbers. ***P < 0.001 when compared with the DMSO control (Ctr) by the Dunnett’s post-hoc test after one-way ANOVA analysis. The shaded areas represent numbers covering 1 or 2 standard deviations (σ) of P5 and DMSO, respectively. **C** Chemical structures of P5 and its derivatives. Relevant carbons in the P5 structure are labelled accordingly

To prevent P5 from further metabolism, we attached F, N_3_, CN, hydroxy or alkoxy groups to all the functional groups at the C-3, C-5, C-6, C-17, C-20 or C-21 positions of the 4-ring backbone of P5 (Additional file 1: Fig. S1). We screened these compounds to find those compounds that promote microtubule polymerization in an in vitro microtubule polymerization assay. In this assay, free fluorescent tubulin was induced to polymerize after incubation with purified CLIP-170 and preformed tubulin seeds at 37 °C. The polymerized fluorescent microtubules were then detected (Fig. 2A) and the images were analyzed. P5 increased the number of polymerized microtubules compared to the control (solvent DMSO) (Fig. 2B). In this in vitro assay, six of approximately 30 newly synthesized compounds promoted microtubule polymerization (Fig. 2B). Two of these P5 analogs, compounds #36 and #43 (Fig. 1C), had microtubule polymerization activity similar to that of P5. Compound #36 has a modification at C-20, while compound #43 has a modification at C-3 (Fig. 2C). Compound #2 (meoP5), an active derivative with a methoxy group in C-3 [34], also increased microtubule polymerization in this assay although its activity was not as robust as that of P5. We chose the most promising derivative, compound #43, for the ensuing experiments.

**Fig. 3:**
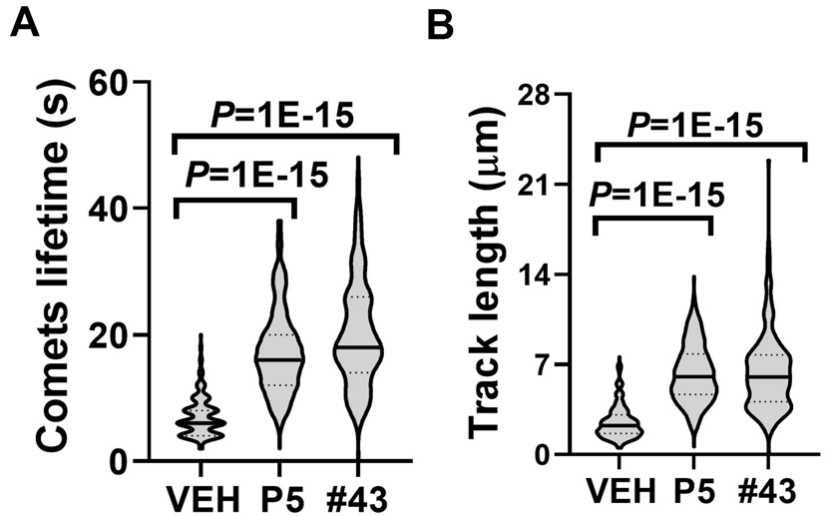
Compound #43 modifies microtubule dynamics in COS1 cells. Microtubule dynamics were measured in CLIP-170-mOrange comets in COS1 cells treated with the steroids, and quantified. P5 and compound #43 both increase **A** comet lifetime and **B** the length of the comet track. *P*-value from one-way ANOVA, Dunnett’s post-hoc test

P5 accelerates microtubule polymerization in live cells through interaction with microtubule “ + TIP”- binding protein CLIP-170 [35]. We tested the effect of compound #43 on microtubule polymerization by measuring microtubule growth in cultured cells (Fig. 3, Additional file 2: Movies S1, Additional file 3: Movie S2, Additional file 4: Movie S3). To visualize microtubule growth, CLIP-170 was fused with the orange fluorescent protein mOrange (CLIP170-mOrange) and expressed in COS1 cells. CLIP-170 is localized to the growing microtubule plus end, appearing as a comet during live imaging [36]. P5 and compound #43 increased CLIP-170 comet life time (Fig. 3A) and length of microtubule tracks (Fig. 3B). These experiments confirm that compound #43 is similar to P5 in its ability to promote microtubule polymerization.

### Compound #43 promotes neurite extension and accelerates the development of cerebellar granule neurons

**Fig. 4:**
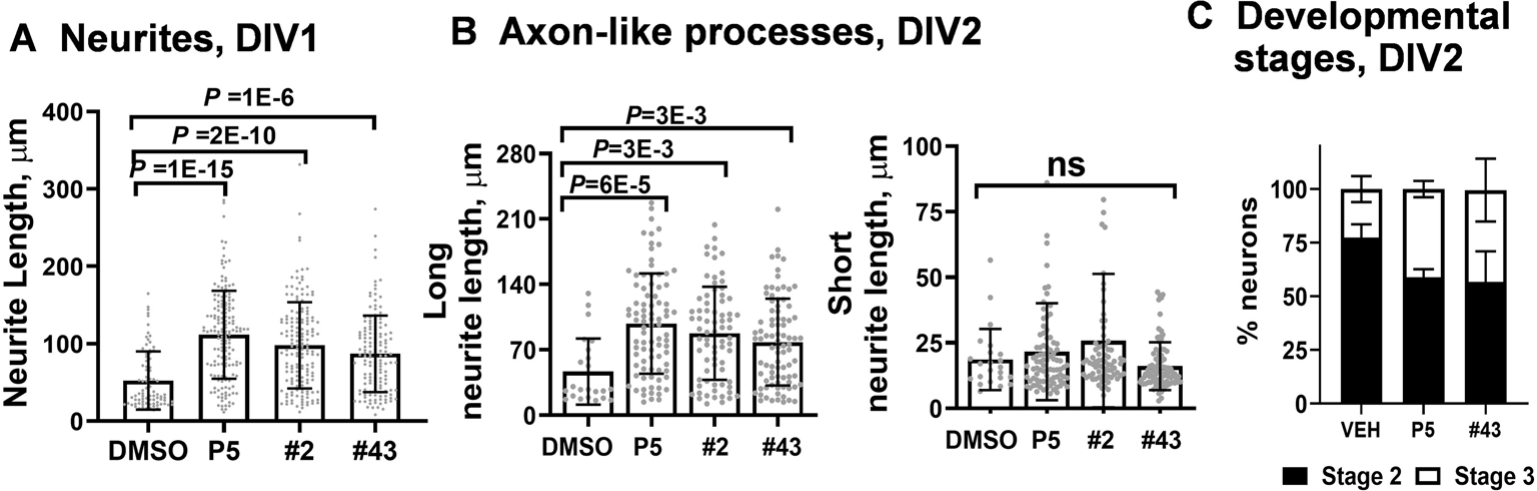
P5 derivatives promote neurite extension and accelerate neuron development in vitro*.* **A** P5 and its derivatives increase neurite outgrowth in cerebellar granule neurons (CGNs) at DIV1. **B** Diverse effects of P5 and its derivatives on the length of axon-like processes in bipolar stage 3 of CGNs. One-way ANOVA with Dunnett’s post-hoc test was used for Panels A and B. **C** P5 and its derivative promote the development of GCNs at DIV 2. The numbers of unipolar stage 2 neurons and bipolar stage 3 neurons were quantified and represented as a percentage across treatments. Chi-square test is the statistical method used for this analysis

Cerebellar granule neurons (CGNs) develop a single neurite on the first day in vitro (DIV 1, stage 2) and develop two axonal-like processes on DIV 2 (stage 3)[37]. Cerebellar granule cell culture is a good model to study neuron development because of its unique morphology with well-defined fate. At DIV1, when neurites started to grow, neurite lengths were increased by treatment with P5, compound #2 or #43 (Fig. 4A). At DIV2, compound #43 also accelerated the growth of fast-growing long axonal-like processes similar to P5 or compound #2 (Fig. 4B). They, however, had no effect on the short neurites (Fig. 4B), which will retract and will be replaced by dendrites during later development [37]. More neurons also advanced to a later stage after treatment with compound #43 at DIV2 (Fig. 4C). We conclude that compound #43 promotes the growth and development of cultured CGNs in a manner similar to P5 and compound #2.

### Compound #43 alters growth cone morphology in cerebellar granule neurons

**Fig. 5:**
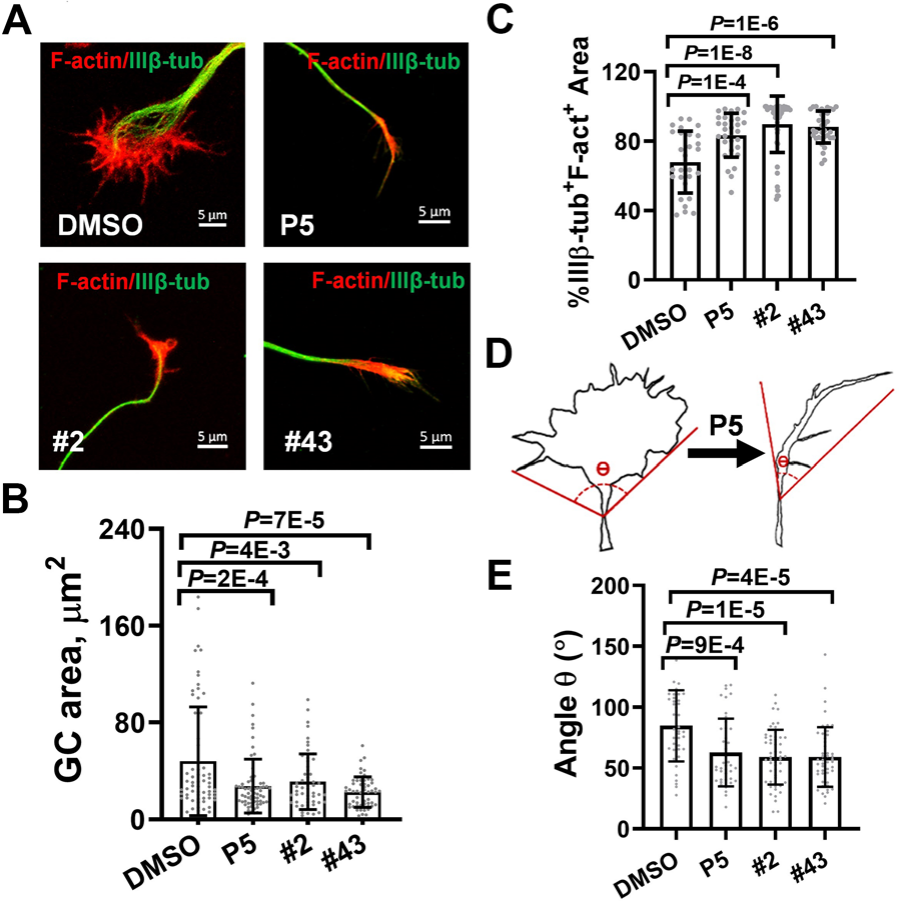
P5 and its derivatives alter growth cone morphology. **A** The growth cone morphology of steroid-treated CGNs at DIV 2 immunostained with rhodamine-phalloidin for F-actin (red) and anti-β-tubulin antibody (green). Scale bar 5 µm. Quantification shows that **B** the total areas of the growth cone are reduced and **C** the percentage of transition zone area represented by the area doubly stained with IIIβ-tub and F-act are increased after treatment with P5 and its derivatives #2 and #43. **D** The scheme of measurement of the growth cone angle. **E** Quantification shows the reduced growth cone angle after neurosteroid treatment. All data were evaluated using one-way ANOVA, Dunnett’s post-hoc test

We examined the effects of P5 derivatives on growth cones of mouse cerebellar granule neuronal culture. The neuronal growth cone oscillates between a fan-shaped, lamellipodial form and a streamlined, filopodial form [38]. Larger fan-shaped forms tend to be associated with a pausing growth cone, while smaller streamlined forms are associated with active axon extension [39]. At DIV 2, the growth cone area was outlined using rhodamine phalloidin and tubulin staining. While growth cones treated with DMSO exhibited a fan shape, those treated with P5, compound #2 and #43 had a more streamlined morphology (Fig. 5A). The area occupied by the growth cones was also smaller after treatment with these compounds (Fig. 5B). The transition zone of the growth cone, defined by the overlap of microtubules with F-actin, was increased (Fig. 5C). We measured the angle between the sides of the growth cone and the endpoint at the end of the neurite shaft (Fig. 5D), and detected smaller angles when cells were treated with P5, compound #2 or #43 (Fig. 5E). The changes in the growth cone induced by P5, compound #2 and #43 are therefore consistent with increased axon extension.

### Compound #43 accelerates the growth of zebrafish cerebellar axons

**Fig. 6:**
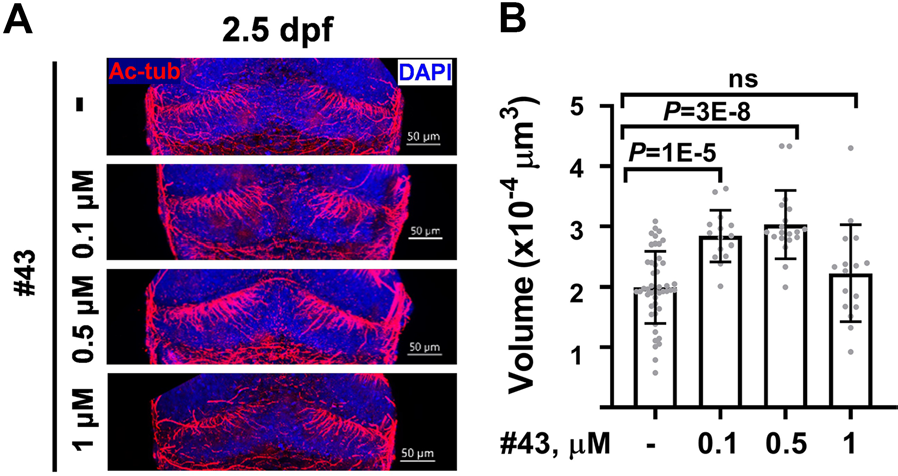
Compound #43 accelerates the formation of zebrafish cerebellum. **A** Zebrafish cerebellum at 60 h postfertilization immunostained with anti-acetylated tubulin antibody (red) and DAPI (blue). Scale bar 50 µm. **B** Increased volume of axonal stable microtubule tracks in developing cerebellum after treatment with compound #43**.** *P*-value from one-way ANOVA, Dunnett’s post-hoc test

To observe the effect of #43 on cerebellum development in vivo, we examined zebrafish cerebellum development after treating embryos with compound #43 from the one-cell stage to day 2.5. Cerebellar axons were immunostained with acetylated tubulin, and the volumes of cerebellar axonal microtubule tracks were quantified. Compound #43 increased the formation of stable microtubule tracks in developing cerebellar axons in a dose-dependent manner, but the effect dropped when the concentration of compound #43 reached 1 µM (Fig. 6). This result indicates that a low concentration of compound #43 is sufficient to accelerate the growth of zebrafish cerebellar axons in vivo.

## Discussion

In this paper, we searched for effective P5 derivatives that can promote microtubule polymerization by synthesizing P5 analogs and screening them. We have identified and characterized compound #43 (3-beta-pregnenolone acetate) that promotes microtubule polymerization and accelerates microtubule dynamics in live cells.

Pregnenolone contains one hydroxyl group at C-3, a double bond at C-5, and one carbonyl at the C-20 position (Fig. 2). The reactivity of steroids depends on the functional groups attached to the tetracyclic backbone [40]. When comparing P5 derivatives with modifications at different carbon positions of the pregnenolone carbon rings, modifications at C-3 blocks the reactivity of the C3-hydroxyl group.

Among all the P5 derivatives that we synthesized, compound #43 is the most effective one that promotes microtubule polymerization (Fig. 2B). Compound #43 with acetoxy-modification at C-3 increased microtubule numbers more efficiently than compound #14 with fluoro-modification. Compounds #43 and #2 are both modified at the C-3 position and have similar effects on neurite outgrowth and neuron development (Fig. 4). Compound #2 (3-beta-methoxy-P5) alleviates the symptoms of degenerative diseases by increasing the dynamic microtubule pool [41]. Here, we show that compound #43 can promote cerebellum development in vivo, and therefore can be further developed as a candidate drug for brain diseases.

In this article, we focus our study on the effect of P5 and #43 in the cerebellum. But the effect of P5 is not limited to cerebellar neurons. It also promotes neurite outgrowth in mouse cortical, hippocampal, and zebrafish forebrain neurons [42]. This is because P5 and #43 enhance microtubule polymerization, and the maturation of microtubule networks is a key process of neuronal development. This notion is consistent with the roles of P5 in enhancing memory [2, 3], reducing chronic pain [4], and alleviating depression [5]. It is possible that #43 will also be beneficial for the development of all neurons.

Axon development in zebrafish cerebellum responds effectively to different concentrations of compound #43 up to 1 µM. At 1 µM of #43, axon development was not higher than that in the control. A similar phenomenon has been observed before; P5 at 20 µM inhibits zebrafish epiboly migration even though P5 at lower concentrations promotes epiboly [43]. In developing axons, microtubules are distributed between dynamic, stable and cold-stable microtubule pools [44]. P5 and compound #43 modify microtubule dynamics in living cells (Fig. 3). The activity of compound #43 in developing axons can change the distribution between dynamic and stable microtubule pools. A high concentration of #43 (1 µM) probably shifts the distribution of the microtubule pools too much, therefore losing beneficial activity for axon development.

## Conclusion

In this aging society, drugs for the treatment of neurological diseases are especially important. Yet very few effective drugs are available partly because of the difficulty to find drugs that penetrate the blood–brain barrier. Steroids have great potential for the brain, because they are lipophilic and get into the brain easily. We found that a pregnenolone derivative, compound #43, accelerates neurite growth and axon formation of cultured neurons. It also stimulates the development of neurons in vivo. We show that compound #43 stabilizes microtubule dynamics and changes growth cone morphology from the paused to fast-growing shape. It is very potent, being functional at the micromolar concentration. Therefore, compound #43 may have the therapeutic potential for the treatment of neurological diseases.

## Materials and methods

All key resources are listed in Table 1.

**Table 1:** Key resources used in this paper

Antibodies	Source	Identifier	Note
Mouse monoclonal anti-acetylated tubulin, clone 6-11B-1	Sigma	Cat#T6793	1:500
Rabbit monoclonal anti-III-beta tubulin [EP1569Y]	Abcam	Cat#ab52623	1:500
Rabbit polyclonal anti-beta tubulin	Proteintech	10094-1-AP	1:200
Goat anti-mouse IgG (H+L) superclonal recombinant secondary antibody, Alexa Fluor 555	Invitrogen	A28180	1:1000
Goat anti-Rabbit IgG (H+L) Highly Cross-Adsorbed Secondary Antibody, Oregon Green 488	Invitrogen	O-11038	1:4000

Experimental models	Source	Identifier	Note
Mouse: C57BL/6J strain	National Applied Research Laboratories		
Zebrafish: TL strain			
COS1 cells			

Plasmids	Source	Identifier	Note
pSC2 + mOrange2-Flag-CLIP1	This paper	N/A	Fluorescent CLIP1 for comet tracking

Steroids	Source	Identifier	Note
Pregnenolone, 5-pregnen-3β-ol-20-one	Sigma-Aldrich	P9129	
P5 derivatives	This paper	N/A	

Reagents for cell culture	Source	Identifier	Note
Dulbecco’s Modified Eagle Medium	Gibco	12100-046	COS1 culture
HyClone Characterized Fetal Bovine Serum	GE Life Sciences	SH30396.03	COS1 culture
Neurobasal Plus Medium	Gibco	A35829-01	Primary neuronal culture
Glutamax-I (100X)	Gibco	35050-061	Primary neuronal culture
B27 Supplement (50X)	Gibco	17504044	Primary neuronal culture
Penicillin–Streptomycin, liquid	Gibco	15140-122	Primary neuronal culture
HEPES, 1 M buffer solution	Gibco	15630-080	Primary neuronal culture
HBSS, 10X	Gibco	14185-052	Primary neuronal culture
Percoll pH 8.5–9.5	Sigma-Aldrich	P1644	Primary neuronal culture
Poly-L-ornithine, 0.01%	Sigma-Aldrich	P4957	Primary neuronal culture
Trypsin–EDTA	Gibco	25300-054	Primary neuronal culture
Trypsin inhibitor from Glycine max (soybean)	Sigma-Aldrich	T6522	Primary neuronal culture
DNase I	Roche	04716728001	Primary neuron harvest

In vitro microtubule polymerization assay	Source	Identifier	Note
Tubulin protein (> 99% pure): porcine brain	Cytoskeleton	T240	
Tubulin protein (rhodamine): porcine brain	Cytoskeleton	TL590M	
GpCpp	Jena Biosciences	NU-405	
Paclitaxel from Taxus brevifolia, ≥ 95% (HPLC), powder	Sigma Aldrich	T7402	
0.5 mL, Open-Top Thickwall Polycarbonate Tube, 8 × 34 mm	Beckman Coulter	343776	

Device	Source	Identifier	Note
Neon Transfection System	Invitrogen	MPK5000	
DeltaVision Core	GE Healthcare	N/A	Live cell imaging for comet tracking
Zeiss LSM710 confocal microscope	Carl Zeiss	N/A	Neurons imaging

Software	Source	Note	
ImarisFileConverter × 64 9.7.2	Oxford Instruments		
Imaris × 64 9.7.2	Oxford Instruments	Quantifying cerebellum volume	
SoftMax Pro 5.3	Molecular Devices		
EnSpire	PerkinElmer		
ImageJ/Fiji	https://imagej.net/Fiji	Base program for MTrackJ	
softWoRx	Applied Precision		
MTrackJ	[47]	CLIP1 comet quantification	
ZEN 2.6	Carl Zeiss	Image processing	
GraphPad Prism 8.0.0	http://www.graphpad.com	Statistical analysis	

Reagents for transfection and staining	Source	Identifier	Note
Neon Transfection System 100 µL Kit	Invitrogen	MPK10096	pSC2+mOrange2-Flag-CLIP1 transfection
DAPI	Life Technologies	D1306	1:10000
Rhodamine phalloidin	Cytoskeleton	PHDR1	
ProLong Gold Antifade Reagent	Invitrogen	P36930	

N/A, not applicable

### Animal models

Male and female C57BL/6JNarl mice were purchased from the National Applied Research Laboratories, Taiwan, and bred at the Animal Facility of the Institute of Molecular Biology, Academia Sinica, under pathogen-free conditions. Animals were maintained at temperature and humidity-controlled conditions under a 14 h:10 h light–dark cycle with free access to food and water, and randomly assigned to experimental groups. Newborn pups were obtained at postnatal day 6 for the preparation of cerebellar granule neuron cultures.

TL strain zebrafish were reared at 28.5 °C under 14 h:10 h light–dark cycle. Embryos were collected 10 min after spawning. The sex of the animals was unknown, as zebrafish sex cannot be determined until approximately 3 weeks post-fertilization [45].

All animal work was approved by the Institutional Animal Care and Utilization Committee of Academia Sinica.

### Plasmid, cell culture and transfection

COS1 cells were grown in DMEM medium supplemented with 10% FBS and maintained at 37 °C in 5% CO_2_. For transfection, COS1 cells were concentrated to a density of 5 × 10^7^ cells/mL and electroporated with the plasmid pSC2 + mOrange2-Flag-CLIP1 through the Neon Transfection System following the electroporation condition recommended by the manufacturer.

Primary cerebellar granule neuronal culture was prepared by obtaining cerebellar tissue in cold Hank’s buffered salt solution HHBS (1xHBSS, 10 mM HEPES, pH = 7.4) from the mouse brain of postnatal day 6 pups. After dissection and the removal of meninges, cerebellar tissue was digested in trypsin–EDTA with DNase I (15 min, 37 °C). Trypsin activity was quenched by incubating the digestion mixture in soybean trypsin inhibitor in HHBS with 0.4% BSA (1 min, 37 °C, 5 min on ice). Cells were dissociated by triturating the mixture with a fire-polished glass pipette and washed in HHBS with 0.4% BSA. The resulting cell suspension was overlaid on 40% Percoll solution and centrifuged (2600 rpm, 10 min, 4 °C) to isolate CGNs from the pellet. Isolated neurons were washed in HHBS with 0.4% BSA, then resuspended in plating media (Neurobasal Plus media with 1X Glutamax-I, 1X B27 Supplement, 1X Penicillin–Streptomycin, 250 µM KCl). Resuspended cells were plated in poly-l-ornithine-coated plastic dishes or glass cover slips and grown in 37 °C in 5% CO_2_.

Steroids were added 1 h after plating of the primary cells or after transfection (for COS1 cells) at a final concentration of 1 µM in 0.01%DMSO and maintained during the whole experiment.

### Immunocytochemistry

CGNs were fixed in 4% paraformaldehyde with 4% sucrose (20 min, 37 °C). Fixed cells were permeabilized in 0.1% Triton X-100 in PBS (5 min, at room temperature (RT) and then incubated in 5% BSA in PBS-Tween 20 (PBST) for antigen blocking (30 min, RT). Primary antibody was added to the cells and allowed to incubate overnight (4 °C). Secondary antibody was added after 3 rinses in PBST and incubated in the dark (2 h, RT). Cells were washed in PBST and then incubated with 14 µM rhodamine phalloidin (10 min, RT). Cell nuclei were stained with DAPI (10 min, RT). The cells were mounted onto glass slides with ProLong Gold Antifade Reagent. Images were taken with a Zeiss LSM710 confocal microscope, using a 63x/1.4 Plan Apochromat objective.

### Immunohistochemistry

In whole zebrafish embryos, immunocytochemistry was performed according to a previously published protocol [46]. Zebrafish embryos at day 2.5 were fixed with 2% trichloroacetic acid in PBS (3 h, RT) and then rinsed thrice in PBS in 0.8% Triton X-100 (PBS-T) for 10 min each. Fixed embryos were dehydrated and rehydrated through graded methanol concentrations (once with 50% MeOH, twice with 100% MeOH, and once with 50% MeOH, 10 min each), rinsed thrice in PBS-T for 5 min each, followed by acetone treatment (20 min, −20 °C) and three rinses in PBS-T, before permeabilization with 10 µg/ml Proteinase K for 3 min. Embryos were refixed in fresh 4% paraformaldehyde in PBS (20 min, RT), rinsed thrice in PBS-T for 10 min each, and then blocked with 10% normal goat serum in PBS-T with 1% dimethyl sulfoxide (PBS-TD) (3 h, RT). The blocked sample was incubated in primary antibody (1:500) overnight at 4° C. Following overnight incubation, embryos were rinsed three times in PBS-T for 30 min each and then incubated in secondary antibody (3 h, RT). Embryos were then washed thrice in PBS-T, stained with DAPI (5 min, RT), then rinsed twice in PBS-T. Images of the zebrafish cerebellum were collected using a Zeiss LSM-710 confocal microscope with an LD C-Apochromat 40x/1.1 W Korr objective, and Z-series stacks were acquired at 1 µm intervals.

### Synthesis of P5 derivatives

All the P5 derivatives were synthesized using P5 as a starting material with the introduction of various functional groups. All reagents were commercially available and used without further purification unless indicated otherwise. All solvents were anhydrous grade unless indicated otherwise. All nonaqueous reactions were carried out in oven-dried glassware under a slight positive pressure of argon unless noted otherwise. Reactions were magnetically stirred and monitored by thin-layer chromatography on silica gel. Flash chromatography was performed on silica gel of 60–200 μm particle size. Yields are reported for spectroscopically pure compounds. Melting points were recorded on a Fargo MP-2D melting point apparatus and are not corrected. ^1^H and ^13^C NMR spectra were recorded on Bruker AV 500 (500 MHz) spectrometer. Chemical shifts are given in *δ* values relative to tetramethylsilane (TMS, δH = 0); coupling constants *J* are given in Hz. Internal standards were CDCl3 (δH = 7.24) for ^1^H NMR spectra, CDCl3 (δC = 77.0) for ^13^C NMR spectra. The splitting patterns are reported as s (singlet), d (doublet), t (triplet), q (quartet), m (multiplet), br (broad), and dd (double of doublets). High-resolution electrospray ionization (EI) mass spectra were conducted on a JMS-700 double focusing mass spectrometer (JEOL, Tokyo, Japan) with a resolution of 8000 (3000) (5% valley definition). The detailed protocols for the synthesis and purification of P5 derivatives are stated in the Additional file 1.

For the treatment of live cells, all steroid compounds were diluted from a 10-mM stock solution in DMSO to a final concentration 1 µM in the appropriate medium. The same final DMSO concentration (0.01%) was used for control treatment. Zebrafish embryos were treated with range of steroid concentrations as indicated below.

### Microtubule polymerization assay

For each steroid treatment, a reaction mixture was made from fluorescent porcine tubulin seed (4 μg/μL porcine tubulin, 833 μM GMPCPP, 333 ng/μL rhodamine tubulin), 30.4 ng FLAG-CLIP-170 and 70 nM steroid in BD buffer. Polymerization was induced at 37 °C for 10 min; premature polymerization was prevented through a short cold incubation (6 min, 4 °C). Subsequent treatment with 0.9% glutaraldehyde (3 min) was used to quench the reaction, and was in turn quenched with 100 μM Tris pH 7. The resulting reaction mixture was spun down on top of a 30% glycerol cushion (75,000 rpm, 17 min, 25 °C) to obtain polymerized tubulin from the pellet. The pellet was resuspended in BD buffer with taxol, and mounted for total internal reflection fluorescence (TIRF) microscopy with Revolution WD. Microtubules were visualized through a 561 nm filter, and imaged at 100X magnification. Microtubules were quatified using an Integrated Morphometry Analysis module in Metamorph.

### Comet tracking

pCS2 +-CLIP170-mOrange transfected COS1 cells were plated on a glass cover slip-overlaid plastic plate at a density of 1150 cells/mm^2^. Cells were allowed to grow for 24 h. The glass cover slip was mounted onto a metal cassette with DMEM in 10% FBS to prepare for live-cell imaging. CLIP170-mOrange comets were imaged in a temperature- and humidity-controlled environment using a DeltaVision Core fluorescence microscope with a 60 × U-APO objective. Images were taken at 2-s intervals for 1 min and deconvoluted with Applied Precision softWoRx imaging software. Comets were and quantified using the MTrackJ plugin software in ImageJ/Fiji.

### Neurite length measurement

The live cerebellar granule neurons, cultured on plastic dishes, were imaged in a temperature and humidity-controlled environment (37 °C, 5% CO_2_) using a Zeiss LSM710 confocal microscope with LD Plan-Neofluar 20x/0.4 Korr Ph2 objective. Images were taken in a 3 × 3 grid with 10% overlap, and stitched together with ZEN 2.6 image processing software. Neurite lengths were quantified by tracing neurites and measuring the length through the region measurement analysis module of MetaMorph.

### Growth cone measurement

The cerebellar granule neurons, stained with IIIβ-tubulin antibody and rhodamine phalloidin for F-actin were imaged with a Zeiss LSM710 confocal microscope, using 63x/1.4 Plan Apochromat objective. The total area of the growth cone and the percentage of the transition zone area of CGNs were defined based on tubulin and F-actin staining using MetaMorph. Growth cone angles were measured by ImageJ.

### Volume cerebellar axon measurement.

Images of Z-serial stacks of the zebrafish cerebellum immunostained with acetylated tubulin antibody were collected using a Zeiss LSM-710 confocal microscope with LD C-Apochromat 40x/1.1 W Korr objective and 1 µm interval. The images were converted by ImarisFileConverter × 64 9.7. The borders of the cerebellum were defined by DAPI staining. The total cerebellar volume of acetylated tubulin in the cerebellum region was analyzed by three-dimensional analysis with Imaris × 64 (Oxford Instruments).

### Quantitation and statistical analysis

GraphPad Prism 8.0.0 was used for data analysis. One-way ANOVA with Dunnett’s post-hoc test was performed on all experiments. All data are expressed as the mean ± standard deviation of the mean and *P* < 0.05 was considered significant. The chi-square test was used for analysis of DIV 2 neuron development when multiple developmental stages were analyzed. Statistical details are indicated in the figures or figure captions.

## Supplementary Information

**Additional file 1:** Additional Materials and Methods and additional **Figure S1.** Structures of P5 and its synthetic derivatives.

**Additional file 2: Movie S1.** The effect of DMSO on microtubule dynamic in COS1 cells, detected by mOrange-CLIP-170.

**Additional file 3: Movie S2.** The effect of P5 on microtubule dynamic in COS1 cells, detected by mOrange-CLIP-170.

**Additional file 4: Movie S3.** The effect of #43 on microtubule dynamic in COS1 cells, detected by mOrange-CLIP-170.

## Data Availability

Not applicable.

## Abbreviations

P5: Pregnenolone
meoP5: 3-Methoxyl-pregnenolone
dpf: Days postfertilization
DIV: Days in vitro
VEH: Vehicle
