# Supplementary material for: A synthetic pregnenolone analog promotes microtubule dynamics and neural development
Source: Cell Biosci. 2022 Dec 1;12:190. doi: 10.1186/s13578-022-00923-2 (PMC9717551; doi:10.1186/s13578-022-00923-2)
Supplement: Supplementary file 1 — Additional file 1: Additional Materials and Methods and additional Figure S1. Structures of P5 and its synthetic derivatives. [file 13578_2022_923_MOESM1_ESM.docx]

**Additional File 1**

This file contains Additional Materials and Methods and Additional Figure S1.

**Additional Materials and Methods.**

All the reagents were commercially available and used without further purification unless indicated otherwise. All solvents were anhydrous grade unless indicated otherwise. All nonaqueous reactions were carried out in oven-dried glassware under a slight positive pressure of argon unless noted otherwise. Reactions were magnetically stirred and monitored by thin-layer chromatography on silica gel. Flash chromatography was performed on silica gel of 60–200 μm particle size. Yields are reported for spectroscopically pure compounds. Melting points were recorded on a Fargo MP-2D melting point apparatus and are not corrected. 1H and 13C NMR spectra were recorded on Bruker AV 500 (500 MHz) spectrometer. Chemical shifts are given in *δ* values relative to tetramethylsilane (TMS, δH = 0); coupling constants *J* are given in Hz. Internal standards were CDCl3 (δH = 7.24) for ^1^H NMR spectra, CDCl3 (δC = 77.0) for 13C NMR spectra. The splitting patterns are reported as s (singlet), d (doublet), t (triplet), q (quartet), m (multiplet), br (broad), and dd (double of doublets). For high-resolution electrospray ionization (EI) mass spectra were conducted on a JMS-700 double focusing mass spectrometer (JEOL, Tokyo, Japan) with a resolution of 8000 (3000) (5% valley definition).

**Synthetic Procedures and Product Characterization.**

**Scheme 1.** Synthesis of pregnenolone (P5) derivatives (**JJS-0011**, **JJS-0043**, **JJS-0140**, **JJS-0357**, **JJS-0359** and **JJS-0360**). DCC = *N*,*N'*-dicyclohexylcarbodiimide; DMAP = 4-dimethylaminopyridine; NBS = *N*-bromosuccinimide.

**3*β*-(4-methylbenzenesulfonyloxy)-5-pregnen-20-one (1)**

To a solution of pregnenolone (5.0 g, 15.7 mmol) in pyridine (30 mL) was added *p*-toluenesulfonyl chloride (10.0 g, 52.1 mmol). After stirring for 20 h at room temperature, the reaction mixture was poured into ice water (100 mL). The white precipitates formed were collected and dried over under reduced pressure to afford the desired product **1** (7.38 g, 99%) as a white solid. C_28_H_38_O_4_S; mp 134‒135 °C; TLC (EtOAc/hexane = 1:3) *R*_f_ = 0.38; ^1^H NMR (CDCl_3_, 500 MHz) δ 7.76 (d, *J* = 8.3 Hz, 2 H), 7.30 (d, *J* = 8.3 Hz, 2 H), 5.27 (dt, *J* = 5.3, 1.8 Hz 1 H), 4.31 (tt, *J* = 11.6, 4.5 Hz, 1 H), 2.48 (t, *J* = 9.0 Hz, 1 H), 2.42 (s, 3 H), 2.27–2.12 (m, 2 H), 2.08 (s, 3 H), 2.01–1.91 (m, 2 H), 1.82–1.77 (m, 4 H), 1.69–1.65 (m, 1 H), 1.64–1.57 (m, 2 H), 1.55–1.47 (m, 2 H,), 143–1.39 (m, 2 H), 1.22–1.07 (m, 2 H), 1.01 (td, *J* = 13.0, 3.5 Hz, 1 H), 0.94 (s, 3 H), 0.93–0.88 (m, 1 H), 0.58 (s, 3 H); ^13^C NMR (CDCl_3_, 125 MHz) δ 209.5, 144.4, 138.8, 134.6, 129.7 (2 ×), 127.5 (2 ×), 123.1, 82.1, 63.5, 56.7, 49.7, 43.8, 38.7, 38.6, 36.8, 36.3, 31.6 (2 ×), 31.4, 28.5, 24.4, 22.7, 21.6, 20.9, 19.1, 13.1; HRMS calcd for C_28_H_38_NaO_4_S [M + Na]^+^: 493.2289, found: *m/z* 493.2291.

**3*β*-(4-Hydroxybutoxy)-5-pregnen-20-one (JJS-0011)**

To a solution of **1** (2.35 g, 5 mmol) in anhydrous 1,4-dioxane (40 mL) was added 1,4-butandiol (4.36 mL, 50 mmol). The reaction mixture was stirred at 110 °C for 48 h, and then concentrated under reduced pressure. The residue was dissolved in CH_2_Cl_2_ (50 mL) and washed with saturated aqueous NaHCO_3_. The aqueous layer was extracted with CH_2_Cl_2_ (50 mL, 3 ×), and the combined organic extracts were washed with brine, dried over MgSO_4_, filtered, and concentrated. The crude product was purified by column chromatography on silica gel (EtOAc/hexane = 1:1) to afford the desired product **JJS-0011** (1.4 g, 77%) as a white solid. C_25_H_40_O_3_; mp 108‒109 °C; TLC (EtOAc/hexane = 1:1) *R*_f_ = 0.45; ^1^H NMR (CDCl_3_, 500 MHz) δ 5.30–5.29 (m, 1 H), 3.61–3.59 (m, 2 H), 3.47 (m, 2 H), 3.13 (tt, *J* = 11.2, 4.3 Hz, 1 H), 2.48 (t, *J* = 9.0 Hz, 1 H), 2.35–2.32 (m, 1 H), 2.18–2.13 (m, 2 H), 2.08 (s, 3 H), 2.02–1.94 (m, 2 H), 1.88–1.81 (m, 2 H), 1.65–1.59 (m, 6 H), 1.58–1.49 (m, 2 H), 1.48–1.39 (m, 4 H), 1.18 (dt, *J* = 11.7, 5.8 Hz, 1 H), 1.13–1.09 (m, 1 H), 1.01 (td, *J* = 13.3, 3.0 Hz, 1 H), 0.95 (s, 3 H), 0.93–0.91 (m, 1 H), 0.58 (s, 3 H); ^13^C NMR (CDCl_3_, 125 MHz) δ 209.5, 140.7, 121.3, 79.0, 67.9, 63.6, 62.6, 56.8, 49.9, 43.9, 38.8, 38.7, 37.1, 36.8, 31.7 (2 ×), 31.4, 30.4, 28.2, 27.3, 24.4, 22.7, 21.0, 19.3, 13.1; HRMS calcd for C_25_H_40_O_3_Na [M + Na]^+^: 411.2870, found: *m/z* 411.2868.

**3*β*-Acetoxy-5-pregnen-20-one (JJS-0043)**

To a cold (0 °C) solution of pregnenolone (4.0 g, 12.6 mmol) in CH_2_Cl_2_ (6 mL) were added pyridine (1.2 mL, 15.75 mmol) and acetic anhydride (1.55 mL, 16.38 mmol). After stirring for 24 h at room temperature, the reaction mixture was concentrated under reduced pressure. The residue was dissolved in EtOAc (100 mL) and washed with saturated aqueous NaHCO_3_. The aqueous layer was extracted with EtOAc (50 mL, 3 ×), and the combined organic extracts were washed with brine, dried over MgSO_4_, filtered, and concentrated. The crude mixture was purified by column chromatography on silica gel (EtOAc/hexane = 1:5) to afford the desired product **JJS-0043** (3.4 g, 76%) as a white solid. C_23_H_34_O_3_; mp 153‒154 °C; TLC (EtOAc/hexane = 1:5) *R*_f_ = 0.37; ^1^H NMR (CDCl_3_, 500 MHz) δ 5.34–5.33 (m, 1 H), 4.57 (tt, *J* = 12.3, 4.4 Hz, 1 H), 2.50 (t, *J* = 9.0 Hz, 1 H), 2.31–2.27 (m, 2 H), 2.17–2.11 (m, 1 H), 2.09 (s, 3 H), 2.00 (s, 3 H), 1.99–1.93 (m, 2 H), 1.86–1.82 (m, 2 H), 1.67–1.61 (m, 2 H), 1.60–1.51 (m, 3 H), 1.49–1.45 (m, 1 H), 1.44–1.41 (m, 2 H), 1.18 (dt, *J* = 11.3, 6.0 Hz, 1 H), 1.16–1.08 (m, 2 H), 0.98 (s, 3 H), 0.97–0.95 (m, 1 H), 0.60 (s, 3 H); ^13^C NMR (CDCl_3_, 125 MHz) δ 209.4, 170.5, 139.6, 122.2, 73.7, 63.6, 56.7, 49.8, 43.9, 38.7, 38.0, 36.9, 36.5, 31.7 (2 ×), 31.5, 27.6, 24.4, 22.7, 21.3, 20.9, 19.2, 13.1; HRMS calcd for C_23_H_34_NaO_3_ [M + Na]^+^: 381.2400, found: *m/z* 381.2406.

**(Pregn-5-en-20-one-3*β*-yloxy)-pent-4-ynoate (JJS-0140)**

To a solution of 4-pentynoic acid (220 mg, 2.25 mmol) in anhydrous THF (20 mL) was added *N*,*N'*-dicyclohexylcarbodiimide (773 mg, 3.75 mmol), 4-dimethylaminopyridine (36 mg, 0.3 mmol) at 0 ^o^C. After stirring for 10 min at room temperature, pregnenolone (474 mg, 1.5 mmol) was added to the reaction mixture. After stirring for 24 h at room temperature, the reaction mixture was filtered to remove the precipitate and the filtrate was concentrated. The residue was purified by column chromatography on silica gel (EtOAc/hexane = 2:8) to afford the desired product **JJS-0140** (460 mg, 77%) as a white solid. C_26_H_36_O_3_; mp 141‒142 °C; TLC (EtOAc/hexane = 2:8) *R*_f_ = 0.29; ^1^H NMR (500 MHz, CDCl_3_) δ 5.35–5.34 (m, 1 H), 4.62 (tt, *J* = 16.3, 4.2 Hz, 1 H), 2.52‒2.45 (m, 5 H), 2.33‒2.26 (m, 2 H), 2.18‒2.11 (m, 1 H), 2.09 (s, 3 H), 2.03‒1.98 (m, 2 H), 1.94 (t, *J =* 2.3 Hz, 1 H), 1.85‒1.82 (m, 2 H), 1.69‒1.59 (m, 3 H), 1.58‒1.49 (m, 2 H), 1.49‒1.45 (m, 1 H), 1.44‒1.42 (m, 2 H), 1.24‒1.17 (m, 1 H), 1.16‒1.10 (m, 2 H), 0.99 (s, 3 H), 0.98‒0.96 (m, 1 H), 0.60 (s, 3 H); ^13^C NMR (125 MHz, CDCl_3_) δ 209.5, 171.1, 139.5, 122.4, 82.5, 74.2, 68.9, 63.6, 56.8, 49.8, 43.9, 38.7, 38.0, 36.9, 36.5, 33.6, 31.7 (x 2), 31.5, 27.7, 24.4, 22.8, 21.0, 19.2, 14.4, 13.2; ESI-HRMS calcd for C_26_H_36_NaO_3_ 419.2562, found: *m/z* 419.2551 [M + Na]^+^.

**3*β*-[4-(Prop-2-yn-1-yloxy)butoxy]-pregn-5-en-20-one (JJS-0357)**

To a solution of **JJS-0011** (70 mg, 0.18 mmol) in anhydrous THF (1.2 mL) was added NaOH (30 mg, 0.72 mmol). After stirring for 15 min at room temperature, the reaction mixture was cooled to 0 ^o^C, and then propargyl bromide (0.03 mL, 0.024 mmol) was slowly added to the reaction mixture. After stirring for 30 h at room temperature, the reaction mixture was diluted with EtOAc (10 mL) and washed with water. The aqueous layer was extracted with EtOAc (20 mL, 3 ×), and the combined organic extracts were washed with 1 N aqueous HCl, saturated aqueous NaHCO_3_ and brine (10 mL). The organic layer was dried over MgSO_4_, filtered, and concentrated. The residue was purified by column chromatography on silica gel (EtOAc/hexane = 2:8) to afford the desired product **JJS-0357** (45 mg, 59%) as a white solid. C_28_H_42_O_3_; mp 82‒83 °C; TLC (EtOAc/hexane = 2:8) *R*_f_ = 0.38; ^1^H NMR (CDCl_3_, 500 MHz) δ 5.30 (dt, *J*  = 5.2, 1.7 Hz, 1 H), 4.10 (d, *J* = 2.3 Hz, 2 H), 3.50 (t, *J* = 6.2 Hz, 2 H), 3.47-3.43 (m, 2 H), 3.10 (tt, *J* = 11.1, 4.5 Hz, 1 H), 2.49 (t, *J* = 9.0 Hz, 1 H), 2.38 (t, *J* = 2.4 Hz, 1 H), 2.35-2.31 (m, 1 H), 2.18-2.11 (m, 2 H), 2.09 (s, 3 H), 2.02-1.94 (m, 2 H), 1.80-1.79 (m, 2 H), 1.60-1.56 (m, 8 H), 1.49-1.38 (m, 4 H), 1.19-1.08 (m, 2 H), 1.04-0.98 (m, 1 H), 0.96 (s, 3 H), 0.95-0.94 (m, 1 H), 0.60 (s, 3 H); ^13^C NMR (125 MHz, CDCl_3_) δ 209.5, 141.0, 121.1, 79.9, 78.8, 74.0, 69.9, 67.6, 63.7, 57.9, 56.9, 50.0, 43.9, 39.1, 38.8, 37.2, 36.8, 31.8, 317, 31.5, 28.4, 26.7, 26.2, 24.4, 22.7, 21.0, 19.3, 13.1; ESI-HRMS calcd for C_28_H_42_NaO_3_ 449.3026, found: *m/z* 449.3018 [M + Na]^+^.

**3*β*-Hydroxy-5-pregnen-7-(but-3-yn-1-yloxy)-20-one (JJS-0359)**

To a solution of **JJS-0043** (717 mg, 2 mmol) in cyclohexane (4 mL) at 70 ^o^C for 20 min was added *N*-bromosuccinimide (534 mg, 3 mmol). The solution was heated at reflux for 2 h and then cooled to room temperature. The reaction mixture was poured into ice water (15 mL) and the formed precipitate was collected through filtration. The crude precipitate was dissolved in CH_2_Cl_2_ and washed with water and brine. The organic layer was dried over MgSO_4_, filtered, and concentrated. The residue was recrystallized from diethyl ether and hexane to afford the desired bromo intermediate **2** (770 mg, 88%) as a yellowish solid. C_23_H_33_BrO_3_; TLC (CH_2_Cl_2_/hexane = 7:3) *R*_f_ = 0.41. The bromo intermediate was used in the next step without further purification.

To a stirring solution of **2** (437 mg, 1 mmol) in anhydrous THF (1.3 mL) were added K_2_CO_3_ (552 mg, 4 mmol), KI (33.2 mg, 0.2 mmol) and 3-butyn-1-ol (1.5 mL, 20 mmol). After stirring for 55 h at room temperature, the reaction mixture was filtered through a pad of Celite with EtOAc. The filtrate was concentrated and the residue was purified by column chromatography on silica gel (EtOAc/hexane = 3:7) to afford the desired product **JJS-0359** (92 mg, 24%) as a white solid. C_25_H_36_O_3_; mp 102‒104 °C; TLC (EtOAc/hexane = 3:7) *R*_f_ = 0.28; ^1^H NMR (CDCl_3_, 500 MHz) δ 5.64 (dd, *J* = 4.9, 1.65 Hz, 1 H), 3.72 (ddd, *J* = 8.9, 7.0, 6.0 Hz, 1 H), 3.58 (tt, 13.3, 2.4 Hz, 1 H), 3.45-3.40 (m, 2 H), 2.56 (t, *J* = 9.2 Hz, 1 H), 2.42-2.38 (m, 2 H), 2.33-2.23 (m, 2 H), 2.18-2.12 (m, 1 H), 2.10 (s, 3 H), 1.98-1.96 (m, 1 H), 1.92 (t, *J* = 2.7 Hz, 1 H), 1.84-1.72 (m, 4 H), 1.70-1.62 (m, 2 H), 1.57-1.50 (m, 2 H), 1.49-1.37 (m, 3 H), 1.21-1.11 (m, 2 H), 0.95 (s, 3 H), 0.58 (s, 3 H); ^13^C NMR (125 MHz, CDCl_3_) δ 209.8, 146.2, 120.9, 81.7, 72.4, 71.3, 68.9, 67.0, 63.5, 49.0, 43.8, 42.4, 42.2, 38.1, 37.3, 37.1, 36.6, 31.5, 31.3, 24.4, 22.8, 20.8, 20.4, 18.2, 12.8; ESI-HRMS calcd for C_25_H_36_NaO_3_ 407.2562, found: *m/z* 407.2555 [M + Na]^+^.

**3*β*-Acetoxy-5-pregnen-7-(but-3-yn-1-yloxy)-20-one (JJS-0360)**

To a solution of **JJS-0359** (80 mg, 0.20 mmol) in anhydrous CH_2_Cl_2_ (1 mL) was added acetic anhydride (0.02 mL, 0.3 mmol), pyridine (0.03 mL, 0.46 mmol) and 4-dimethylaminopyridine (2 mg, 0.01 mmol) at 0 ^o^C. After stirring for 8 h at room temperature, the reaction mixture was diluted with EtOAc (50 mL), and then washed with water, 1 N aqueous HCl and brine. The aqueous layer was extracted with EtOAc (20 mL, 3 ×) and the combined organic extracts were dried over MgSO_4_, filtered, and concentrated. The residue was purified by column chromatography on silica gel (EtOAc/hexane = 2:8) to afford the desired product **JJS-0360** (42 mg, 50%) as a white solid. C_27_H_38_O_4_; mp 161‒164 °C; TLC (EtOAc/hexane = 3:7) *R*_f_ = 0.36; ^1^H NMR (CDCl_3_, 500 MHz) δ 5.67‒5.66 (m, 1 H), 4.65 (tt, *J* = 12.2, 4.3 Hz, 1 H), 3.72 (ddd, *J* = 12.9, 8.9, 6.1 Hz, 1 H), 3.43‒3.37 (m, 2 H), 2.56 (t, *J* = 9.2 Hz, 1 H), 2.41‒2.38 (m, 2 H), 2.35‒2.32 (m, 2 H), 2.17‒2.12 (m, 1 H), 2.09 (s, 3 H), 2.01 (s, 3 H), 1.98‒1.95 (m, 1 H), 1.92 (t, *J* = 2.6 Hz, 1 H), 1.86‒1.80 (m, 2 H), 1.79‒1.64 (m, 3 H), 1.59‒1.54 (m, 2 H), 1.49‒1.40 (m, 4 H), 1.21‒1.15 (m, 2 H), 0.96 (s, 3 H), 0.58 (s, 3 H); ^13^C NMR (125 MHz, CDCl_3_) δ 209.7, 170.4, 145.1, 121.8, 81.8, 73.3, 72.1, 68.8, 66.9, 63.5, 49.0, 43.7, 42.3, 38.1, 38.0, 37.4, 37.0, 36.4, 31.5, 27.4, 24.3, 22.8, 21.3, 20.7, 20.3, 18.1, 12.8; ESI-HRMS calcd for C_27_H_38_NaO_4_ 449.2668, found: *m/z* 449.2662 [M + Na]^+^.


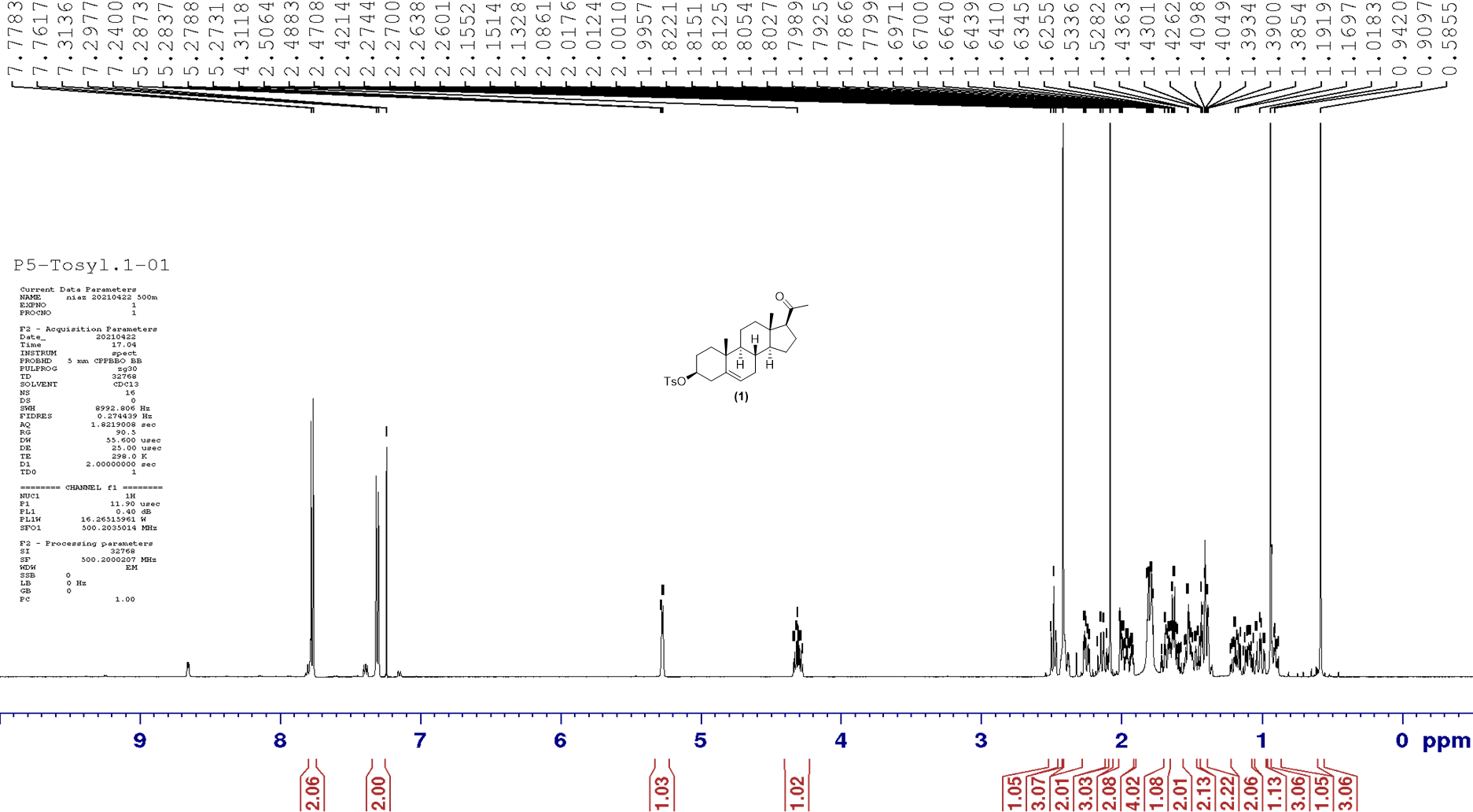


^1^H NMR spectrum of **1** (CDCl_3_, 500 MHz)


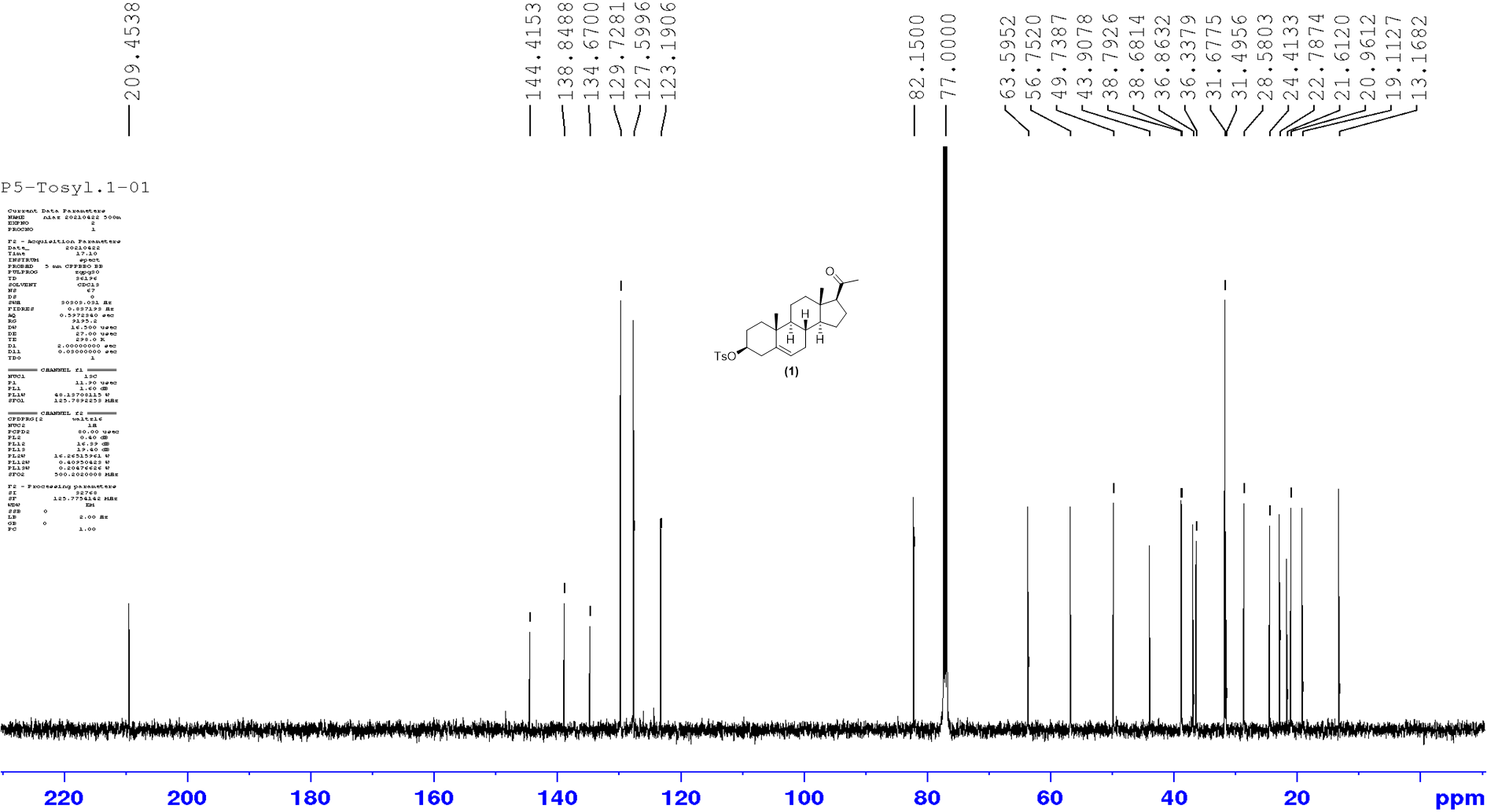


^13^C NMR spectrum of **1** (CDCl_3_, 125 MHz)


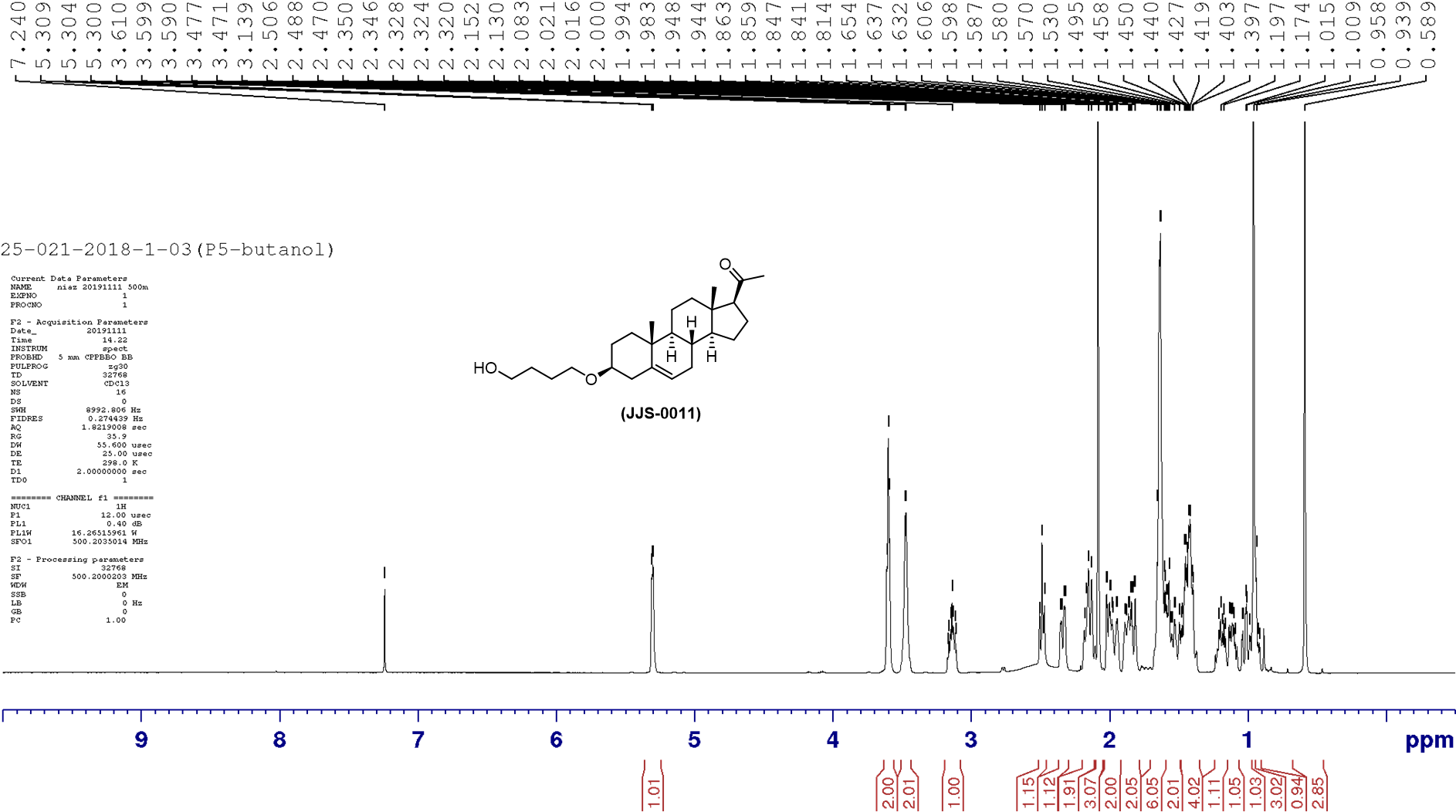


^1^H NMR spectrum of **JJS-0011** (CDCl_3_, 500 MHz)


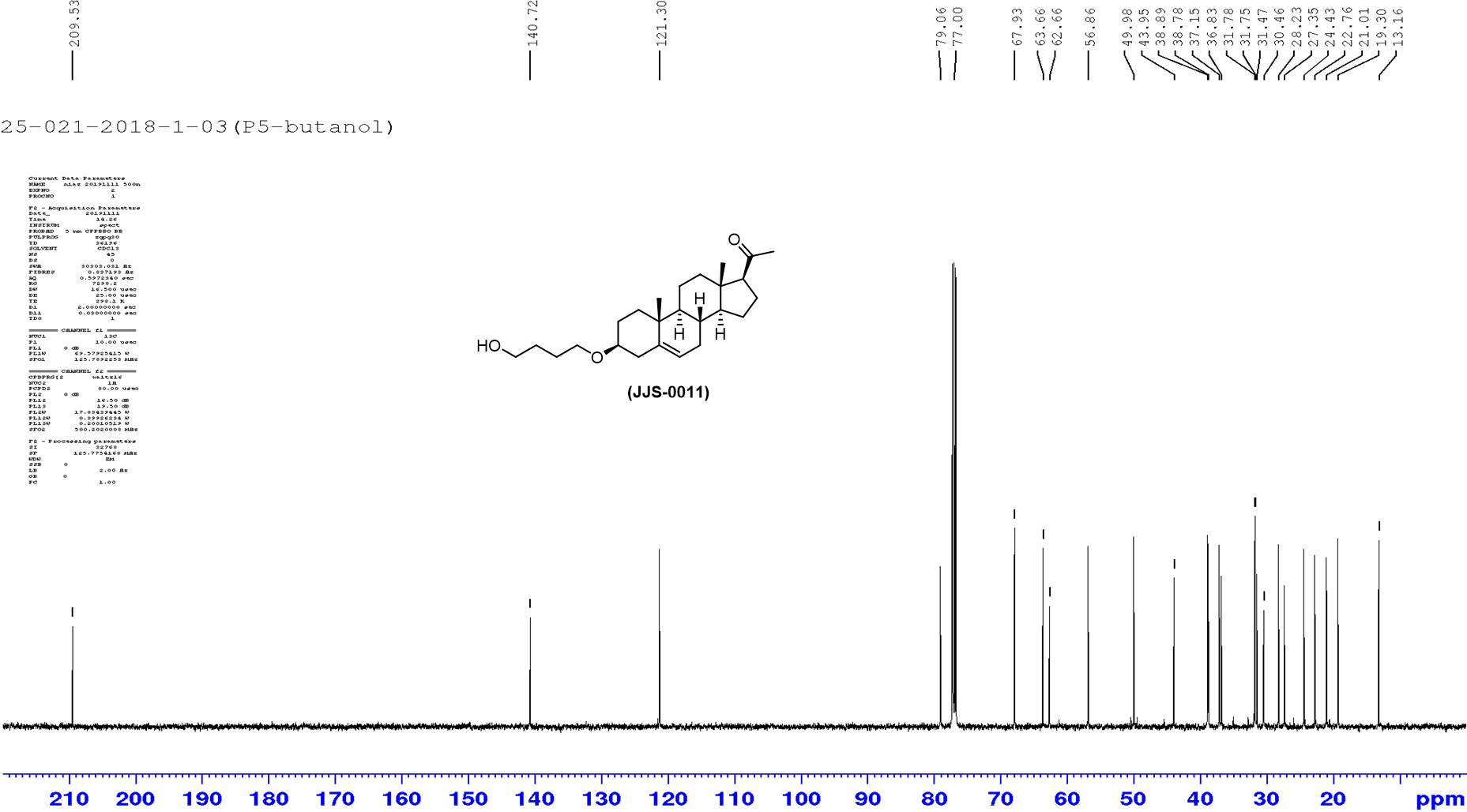


^13^C NMR spectrum of **JJS-0011** (CDCl_3_, 125 MHz)

^
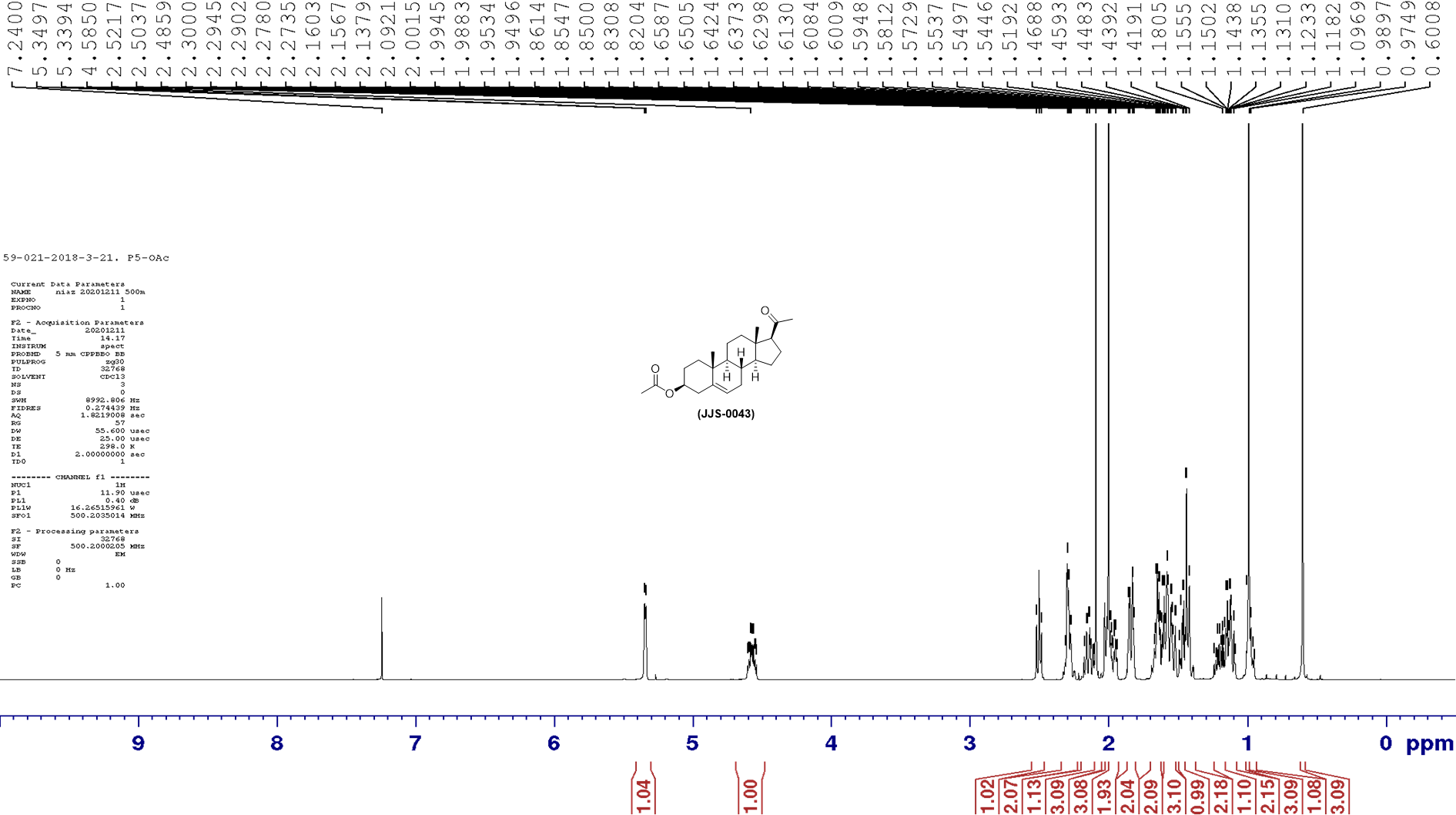
^

^1^H NMR spectrum of **JJS-0043** (CDCl_3_, 500 MHz)


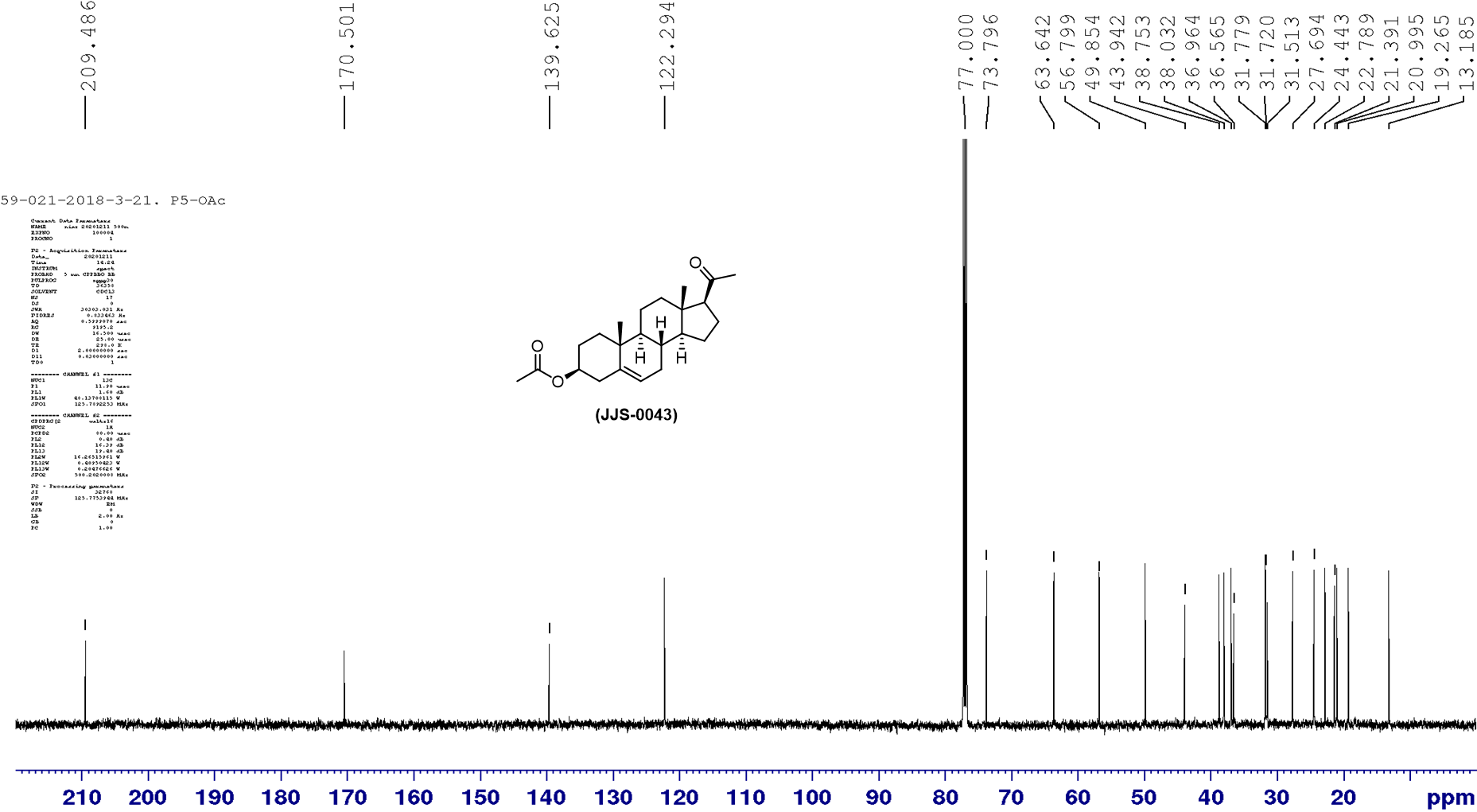


^13^C NMR spectrum of **JJS-0043** (CDCl_3_, 125 MHz)


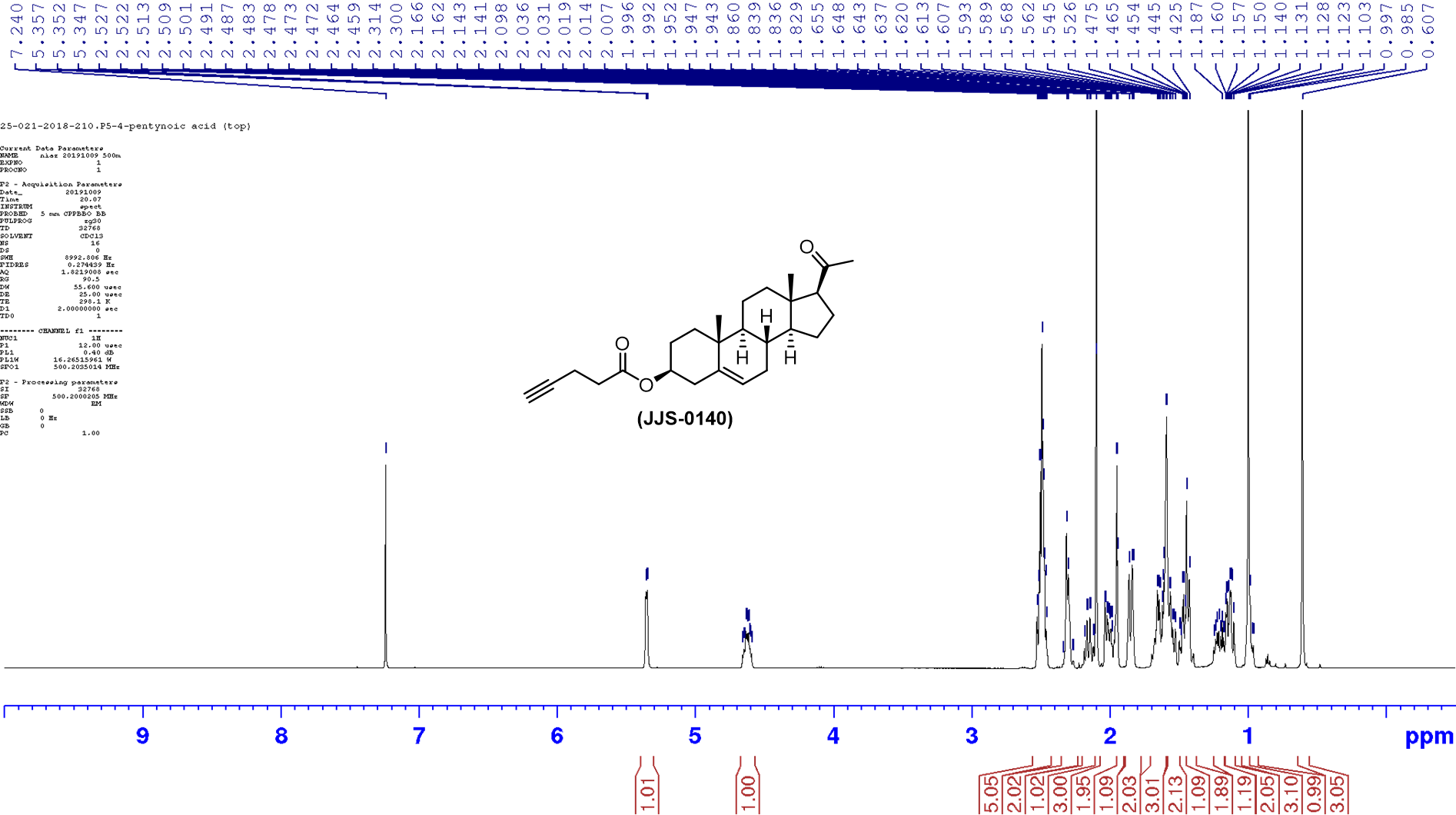


^1^H NMR spectrum of **JJS-0140** (CDCl_3_, 500 MHz)


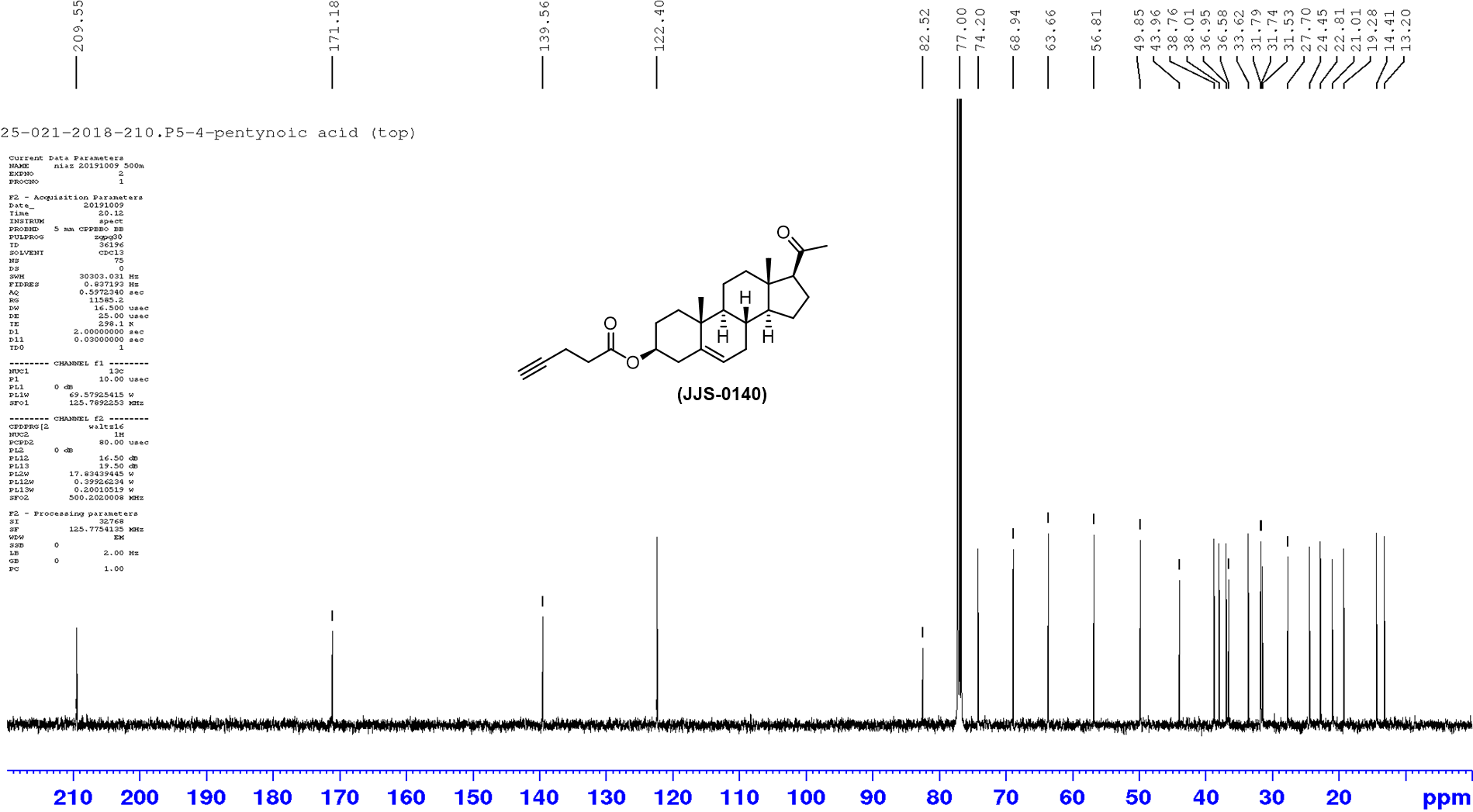


^13^C NMR spectrum of **JJS-0140** (CDCl_3_, 125 MHz)


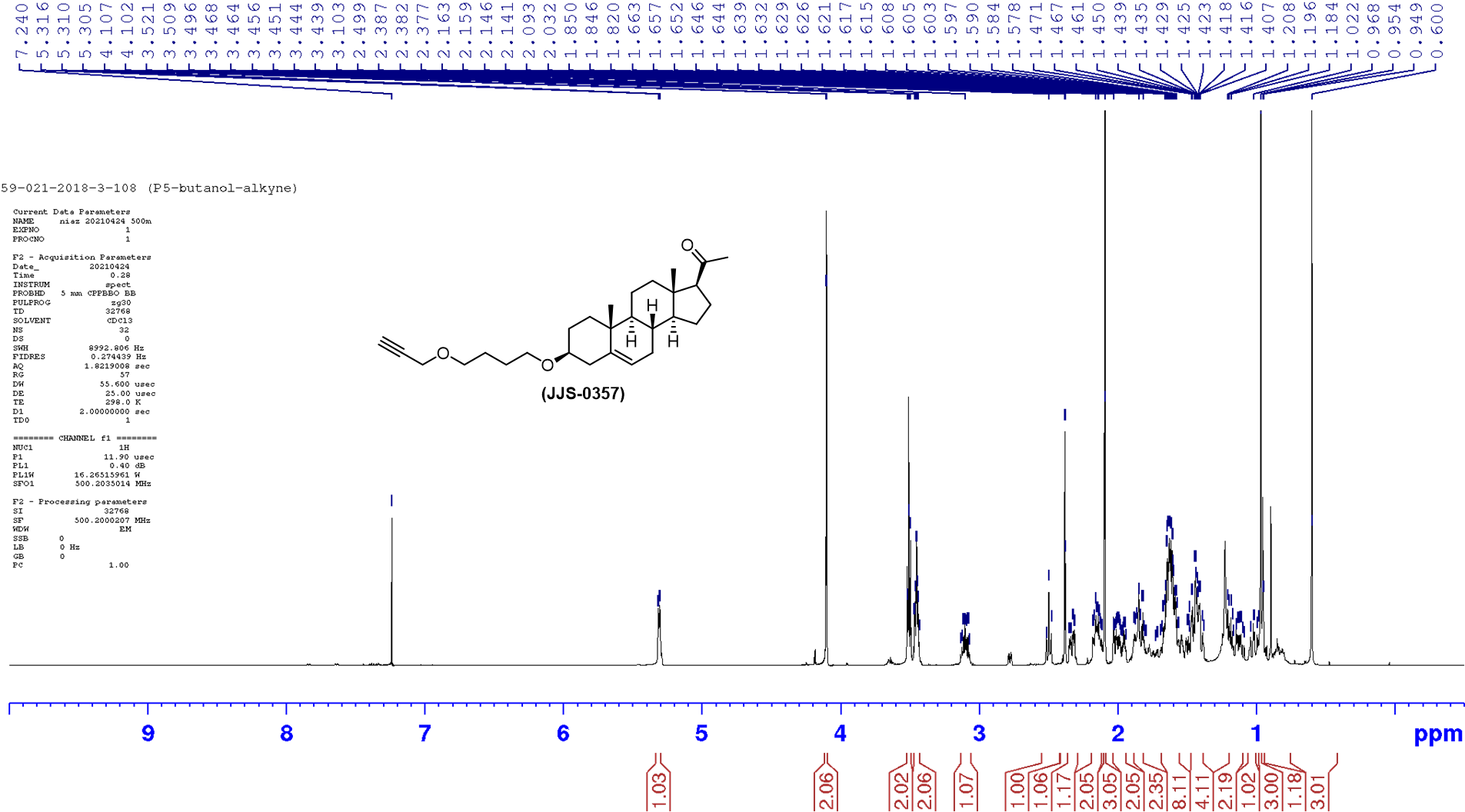


^1^H NMR spectrum of **JJS-0357** (CDCl_3_, 500 MHz)


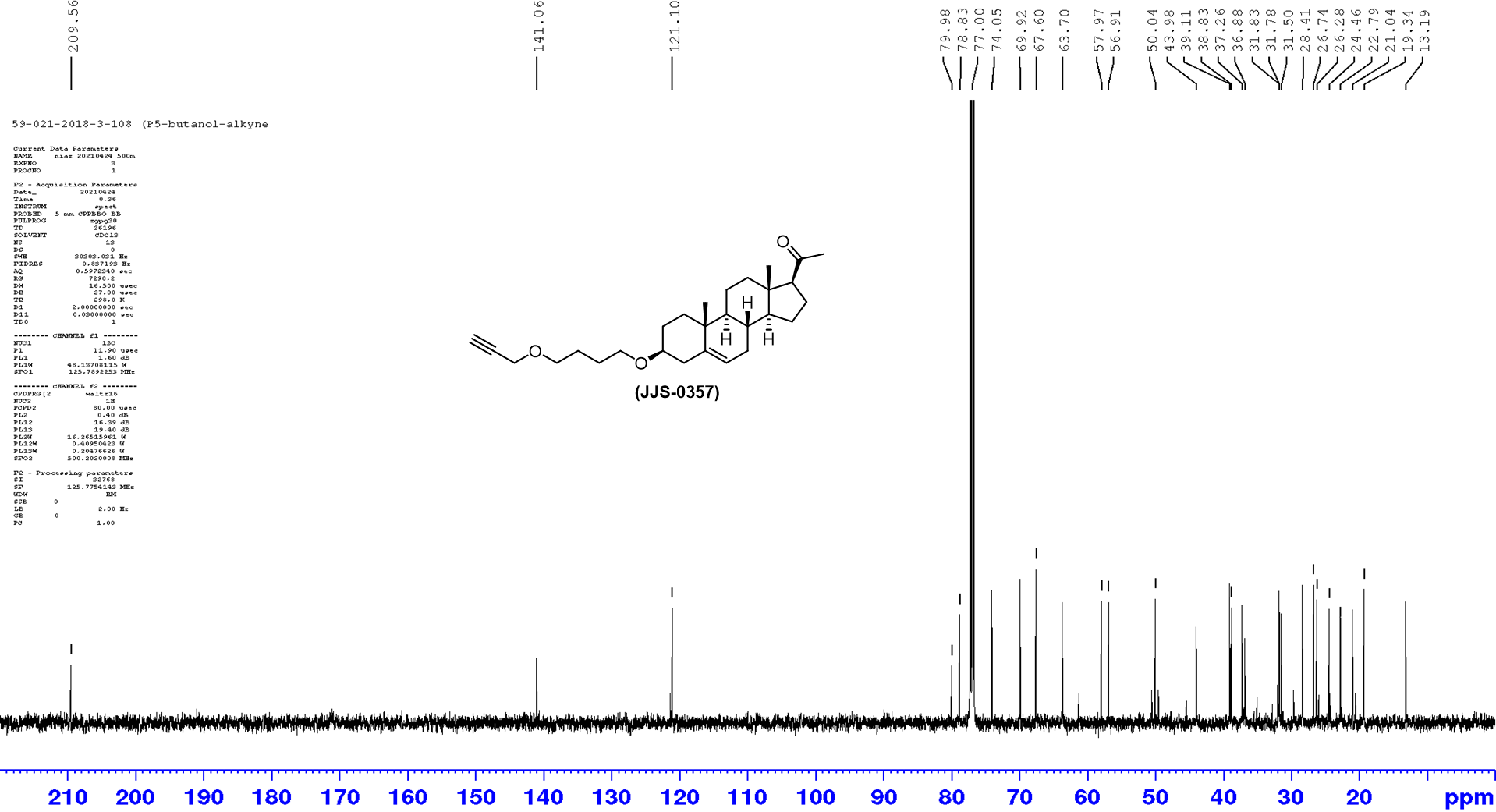


^13^C NMR spectrum of **JJS-0357** (CDCl_3_, 125 MHz)


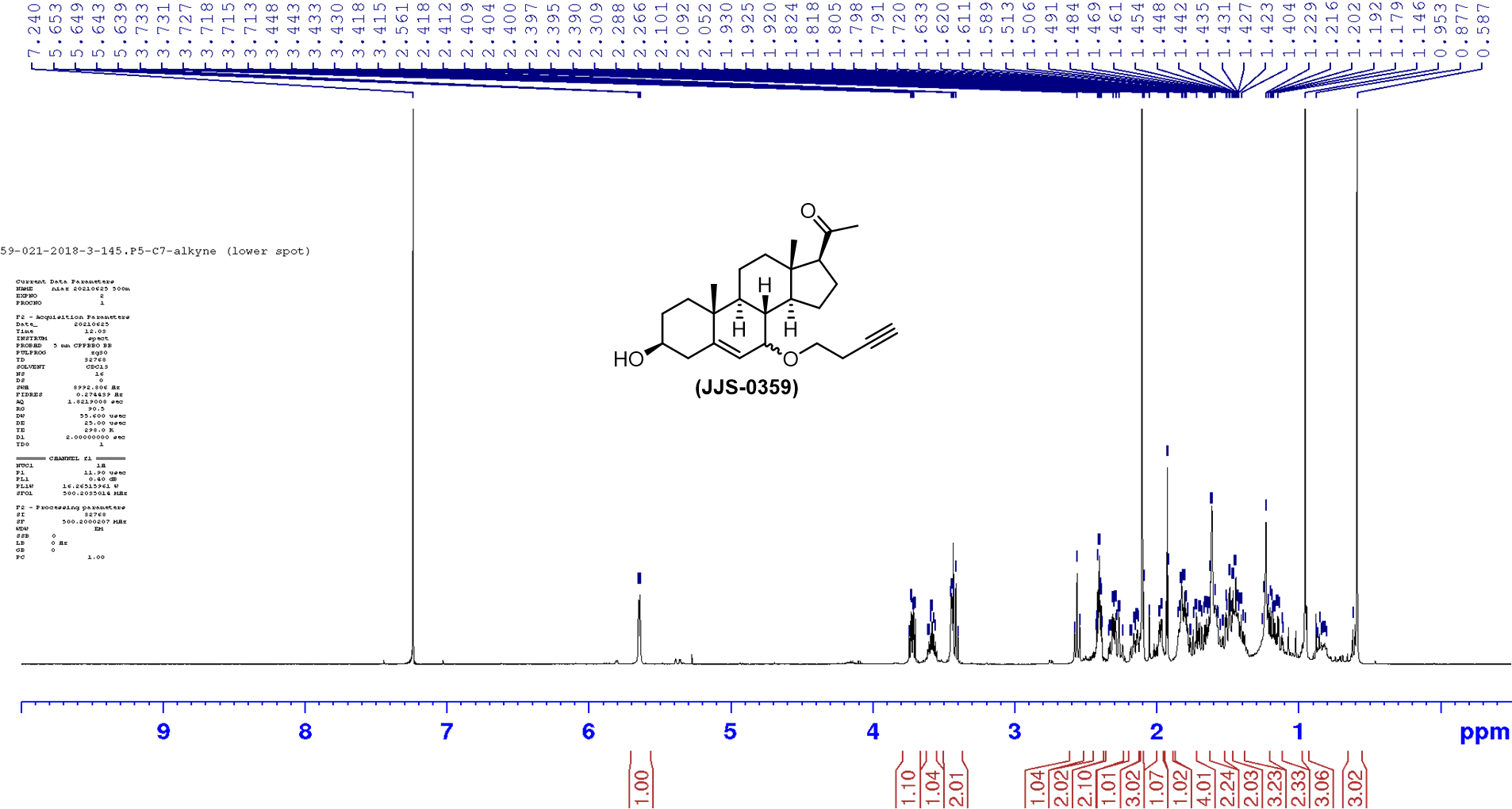


^1^H NMR spectrum of **JJS-0359** (CDCl_3_, 500 MHz)


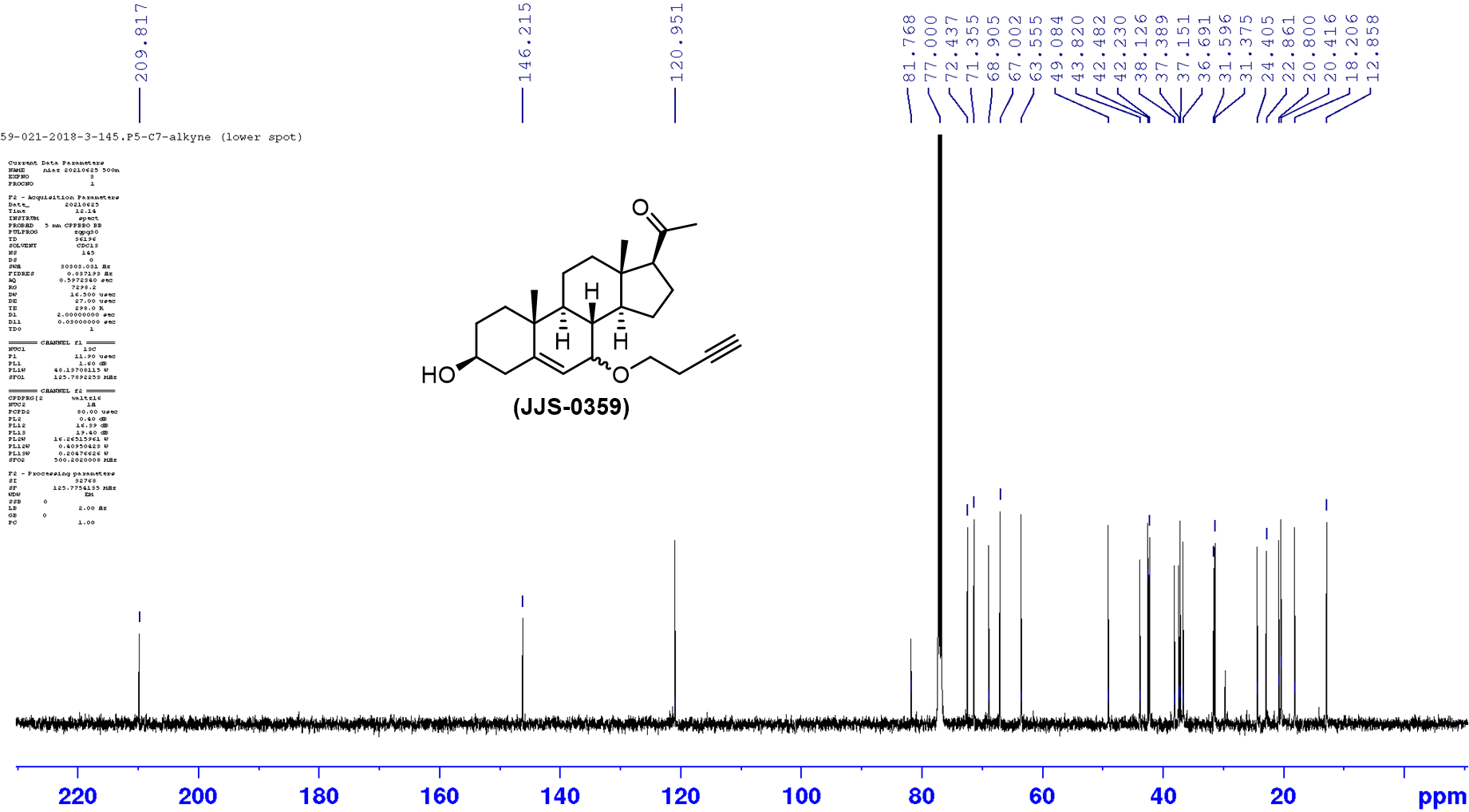


^13^C NMR spectrum of **JJS-0359** (CDCl_3_, 125 MHz)


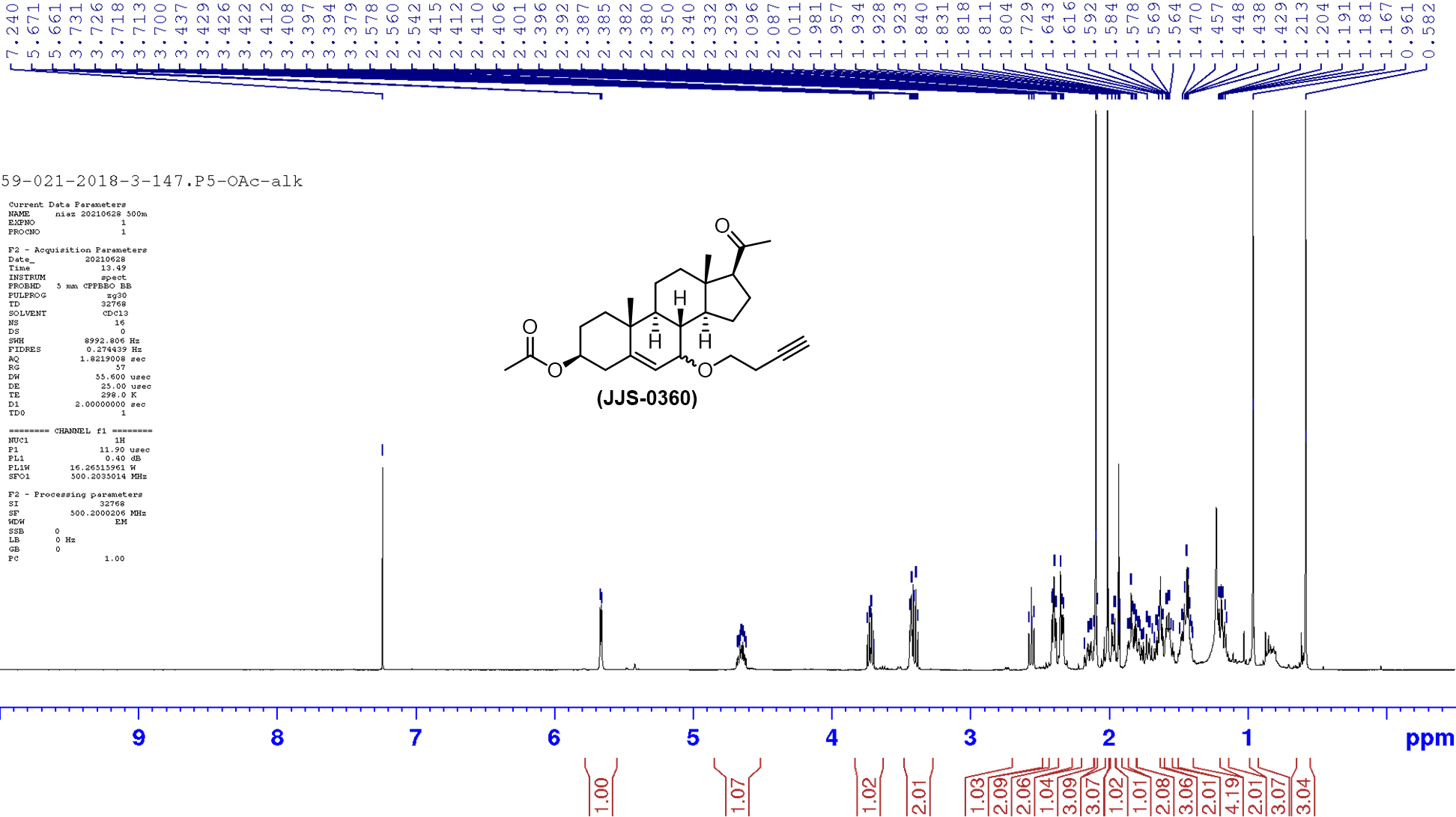


^1^H NMR spectrum of **JJS-0360** (CDCl_3_, 500 MHz)


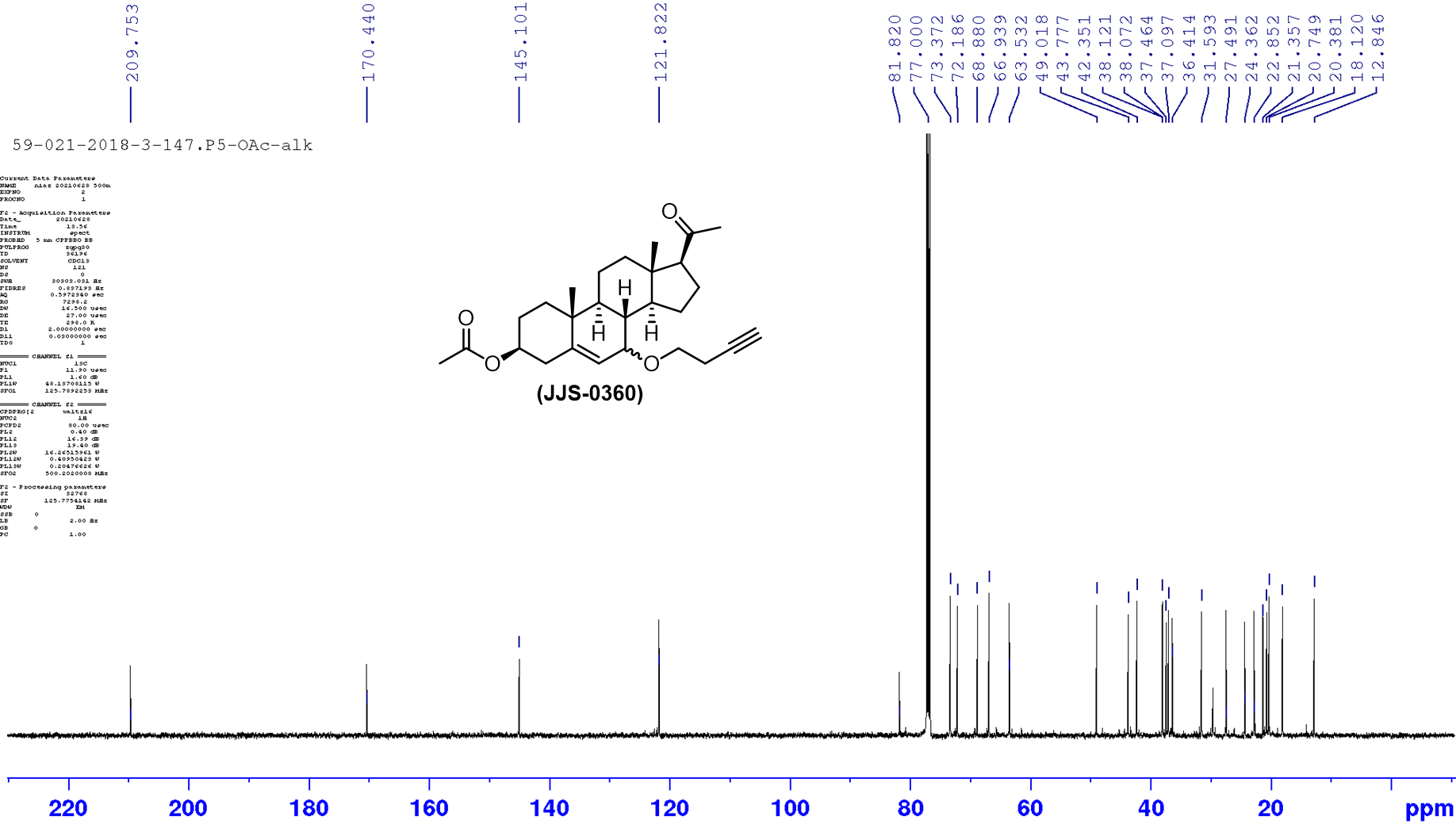


^13^C NMR spectrum of **JJS-0360** (CDCl_3_, 125 MHz)

**Additional Fig. S1. Structures of P5 and its synthetic derivatives.**
